# Bioproduced Nanoparticles Deliver Multiple Cargoes via Targeted Tumor Therapy In Vivo

**DOI:** 10.1021/acsomega.4c03277

**Published:** 2024-07-23

**Authors:** Parastoo Pourali, Eva Neuhöferová, Volha Dzmitruk, Milan Svoboda, Eva Stodůlková, Miroslav Flieger, Behrooz Yahyaei, Veronika Benson

**Affiliations:** † Institute of Microbiology, Czech Academy of Sciences, Praha 142 20, Czech Republic; ‡ Center of Molecular Structure, Institute of Biotechnology, Czech Academy of Sciences, Vestec 252 20, Czech Republic; § Institute of Analytical Chemistry, Czech Academy of Sciences, Brno 602 00, Czech Republic; ∥ Department of Medical Sciences, Shahrood Branch, Islamic Azad University, Shahrood 9WVM+5HC, Iran; ⊥ Department of Medical Sciences, Biological Nanoparticles in Medicine Research Center, Shahrood Branch, Islamic Azad University, Shahrood 9WVM+5HC, Iran; # Faculty of Health Studies, Technical University of Liberec, Liberec 46001, Czech Republic

## Abstract

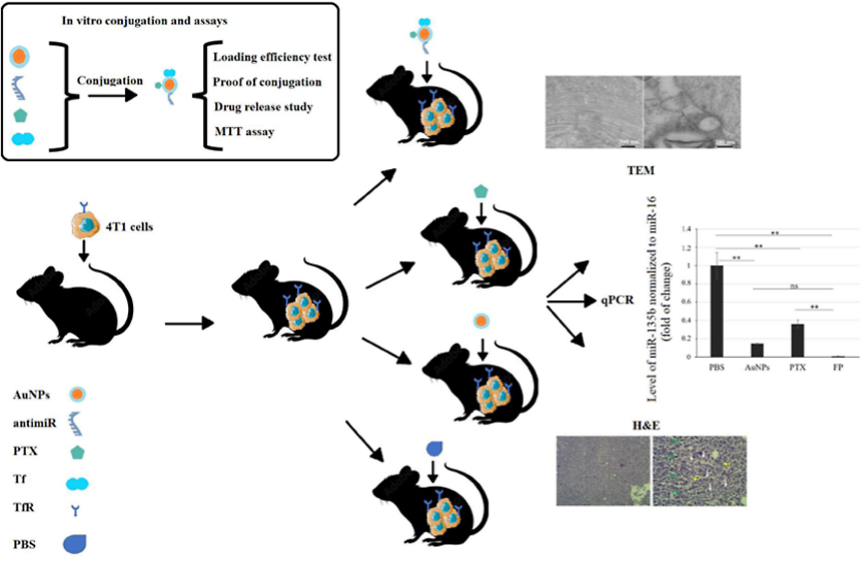

This study recognized biologically produced gold nanoparticles (AuNPs) as multiple cargo carriers with a perspective of drug delivery into specialized tumor cells in vivo. Paclitaxel (PTX), transferrin, and antimiR-135b were conjugated with AuNPs and their uptake by mouse tumor cells in an induced breast cancer model was investigated. Each of the above-mentioned molecules was conjugated to the AuNPs separately as well as simultaneously, loading efficiency of each cargo was assessed, and performance of the final product (FP) was judged. After tumor induction in BALB/c mice, sub-IC_50_ doses of FP as well as control AuNPs, PTX, and phosphate buffered saline were administered in vivo. Round AuNPs were prepared using *Fusarium oxysporum* and exhibited a size of 13 ± 1.3 nm and a zeta potential of −35.8 ± 1.3 mV. The cytotoxicity of individual conjugates and FP were tested by MTT assay in breast tumor cells 4T1 and nontumor fibroblasts NIH/3T3 cells. The conjugation of individual molecules with AuNPs was confirmed, and FP (size of 54 ± 14 nm and zeta potential of −31.9 ± 2.08 mV) showed higher 4T1-specific toxicity in vitro when compared to control conjugates. After in vivo application of the FP, transmission electron microscopy analyses proved the presence of AuNPs in the tumor cells. Hematoxylin and eosin staining of the tumor tissue revealed that the FP group exhibited the highest amounts of inflammatory, necrotic, and apoptotic cells in contrast to the control groups. Finally, qPCR results showed that FP could transfect and suppress miR-135b expression in vivo, confirming the tumor-targeting properties of FP. The capacity of biologically produced gold nanoparticles to conjugate with multiple decorative molecules while retaining their stability and effective intracellular uptake makes them a promising alternative strategy superior to current drug carriers.

## Introduction

Breast cancer (BC) is one of the most common cancers in women^1^ as there were 2.3 million new BC cases (with 685,000 deaths) reported worldwide in 2020.^2^ There are several different strategies to combat BC depending on the subtype and stage of the cancer.^1,3^ Surgical resection, chemotherapy, and radiation therapy are the three main therapies used to treat BC, and each has its own advantages and disadvantages. For example, radiation therapy affects the surrounding normal tissue. Both chemotherapy and radiation therapy cause DNA damage. Although chemotherapy drugs target fast-growing cells and suppress the proliferation of cancer cells, in some cases, resistance to the anticancer drugs occurs. Therefore, there is a need for a new approach that allows us to overcome these issues.^4^

Through many efforts in these decades, the characteristics and pathways of different subtypes of BC have been identified, which has helped to better target a specific subtype of BC.^3,5^ Recently, new strategies for BC treatment focus on targeted therapies. The goal of the targeted therapy is to deliver anticancer drugs into selective cancer cells without affecting the noncancerous environment. For example, trastuzumab therapy (Herceptin, an IgG1 monoclonal humanized mouse antibody) binds to the extracellular domain (ECD) of the HER2 transmembrane receptor. Blockade of the receptor inhibits the downstream signal transduction pathway and induces apoptosis.^5^ A newer strategy is receptor-targeted therapy.^6^ In this method, the specific chemotherapeutic agent is bound to the specific ligand of the cancer cell, and the drug can enter the cells by binding the ligand to its receptor. After receptor-mediated endocytosis, the drug is delivered into the cancer cells.^6^ For example, the ado-trastuzumab emtansine conjugate consisting of a fungal toxin emtansine (DM1) and trastuzumab; in this approach, trastuzumab, possessing affinity to HER2, mediates conjugate internalization and delivery of DM1 into tumor cells, resulting in microtubules blockage.^5^ Application of receptor-targeted therapy allows a decrease in dosage of the chemotherapeutic drugs, their cumulative toxicity, and side effects for patients.

Although this technique has its own advantages, tumor resistance has been reported in some cases. One type of resistance is called acquired resistance.^7^ In tumors, there are heterogeneous populations of both types, target-positive and target-negative, cancer cells. The administration of targeted therapy triggers a selection pressure that leads to the selection of target-negative tumor cells that do not respond to the therapy. This is the main reason for acquired cancer resistance development.^7,8^ Overcoming or preventing this problem can be addressed by multiple targets strategies with a synergistic antitumor effect aiming at different subtypes of cancer cells.^7^ Using multiple targets strategies prior to the development of acquired tumor resistance may prevent treatment complications caused by regrowth of a drug-resistant tumor.

These days, molecules used for the multiple targeting strategy include specific cancer cell ligands (such as monoclonal antibodies, hormones, or small protein molecules), ribonucleic acid interference (RNAi) molecules, and chemotherapeutic agents.^9^ The use of RNAi molecules to inhibit gene expression in cancer cells is straightforward, but one of the drawbacks of this technique is the short half-life of the interfering RNA. It has been reported that the association of these molecules with some types of nanoparticles (NPs) increases their plasma circulation time and payload to the cancer cells without affecting the noncancer cells.^10^ In this strategy, the NPs protect the RNAs from RNases and deliver the RNA usually combined with a cytostatic directly into the cytoplasm of the cancer cells without the need for an additional transfection reagent. Among the various NPs used for this objective, gold nanoparticles (AuNPs) are attracting more attention due to their biocompatibility and ability to bind to various molecules.

There are three different techniques for producing AuNPs, which are divided into biological, physical, and chemical techniques. The disadvantages of the chemical method for producing AuNPs are the persistence of some toxic elements on the surfaces of the produced AuNPs and their environmental toxicity.^11^ The disadvantages of the physical technique are its time-consuming preparation and polydisperse product. The production of AuNPs by biological systems is safe, simple, fast, inexpensive, and environmentally friendly.^11,12^ Different types of microbial strains, such as bacteria and fungi, are capable of producing high yields of AuNPs. In principle, the microorganisms reduce the gold ions to AuNPs to reduce their toxicity. This reduction occurs intracellularly or extracellularly and via nonenzymatic or enzymatic pathways.^13,14^ After the reduction of gold ions, molecules (like proteins) secreted by the microorganisms naturally cover the surface of the newly formed AuNPs. These molecules are called capping agents, and they prevent AuNPs from agglomerating, even when the produced AuNPs are in close contact.^15,16^ Therefore, the obtained AuNPs are always monodisperse and uniform in size. These capping agents are responsible for direct conjugation of the AuNPs with other molecules through electrostatic or ionic interactions due to the charged amino acids on their surface,^15,17^ which eliminates the need for additional linkers.^17−20^ AuNPs produced by the biological method possibly evade the immune system,^21^ and there are some reports on their nonimmunogenic nature.^22^

In this study, AuNPs were prepared using a biological method. They were then conjugated with different molecules to produce a synergistic effect for multifunctional targeted drug delivery. Paclitaxel (PTX, as the BC chemotherapeutic drug), antimiR-135b (for targeting microRNA-135b), and human transferrin (Tf, as a ligand for its receptor overexpressed on BC cells) were conjugated to AuNPs. MicroRNA-135b (miR-135b) was chosen following our past research.^23^ The miR-135b is overproduced in BC cells acting as an oncogene and regulator of BC cell proliferation, migration, and invasion.^24^ Down-regulation of miR-135b was shown to be a possible therapeutic strategy for an adjuvant treatment of BC.^25^ After conjugation of the individual components with biologically produced AuNPs, we tested the performance of the different AuNP conjugates in vitro. Subsequently, we followed with administration of the multicomponent AuNP conjugate as well as control AuNPs and PTX into tumor-bearing animals in vivo. We found the perspective performance of the multicomponent AuNP conjugate regarding tumor cells internalization, miR-135b knockdown, and tumor cells death. The aim of our study was to investigate a multiple decoration of the gold NPs representing a more flexible and effective approach. Targeting structure (here, Tf) eases and specifies conjugate internalization primarily into cancer cells. Both, PTX and antimir-135b, are effector molecules that act in different ways (for example, apoptosis induction vs invasion block), and their synergism results in complex anticancer effect.

## Materials and Methods

### Cultivation of Fungi and Production of AuNPs

*Fusarium oxysporum* (CCF 3732) was cultured in Sabouraud Dextrose Broth (SDB, Sigma-Aldrich, Prague, Czech Republic) at 30 °C under shaking conditions for approximately 1 week. The biomass was separated from the culture medium by centrifugation at 8000*g* for 15 min, and the supernatant was used to prepare extracellular AuNPs.^17^ For this purpose, HAuCl_4_.3H_2_O (Sigma-Aldrich, Prague, Czech Republic) was added to the supernatant at a final concentration of 1 mmol, and the pH was adjusted to 7.5. The dispersion was heated directly at 80 °C for 5 min, and the color change indicated the formation of AuNPs. The negative control flask containing sterile SDB and a final concentration of 1 mmol HAuCl_4_.3H_2_O was heated under the same conditions.

### Characterization of AuNPs

Prior to any test, the obtained color-changed AuNPs dispersions were washed three times with ddH_2_O. During the washing process, the AuNPs were collected by centrifugation at 15,000*g* for 30 min^11^ AuNPs were characterized using spectrophotometry, transmission electron microscopy (TEM), energy-dispersive X-ray spectroscopy (EDS), zetasizer, and Fourier-transform infrared spectroscopy (FTIR); and the methodologies were described previously.^15,16^ Au concentration was determined by graphite furnace-atomic absorption spectroscopy (GF-AAS) as described in our previous study.^15^

### Conjugation and Loading Efficiency Tests

Different molecules (Tf, antimiR-135b, and PTX) were used for the conjugation with AuNPs. First, a standard curve was constructed based on the known amounts of each molecule (Figure S1), and then each molecule at different amounts was conjugated separately. The best conjugate with the highest loading but still stable (according to the color change of AuNPs and spectrophotometry analysis) was selected, and its loading efficiency on AuNPs was measured. Using the equation from each curve and an online tool (available at www.wolframalpha.com), the amount of bound molecules was determined. Finally, AuNPs-Tf- antimiR-135b-PTX (i.e., final product (FP)) was prepared, and the amount of each molecule was determined. All conjugation experiments were performed in triplicate.

### Conjugation of AuNPs to Tf and Tf-Load Assessment

Eight μL of Tf-labeled Texas Red (5 mg/mL, Life Technologies, Prague, Czech Republic) stock solution was diluted with 192 μL of RNase-free ddH_2_O. To generate a Tf standard curve, 1/2 serial dilutions of Tf were prepared in a 96-well microtiter plate using RNase-free ddH_2_O, and the fluorescence intensity of the wells was determined at excitation/emission wavelengths of 580/609 nm using a Tecan spectrophotometer (Thermo Fisher Scientific, Prague, Czech Republic).

For the conjugation study, 32 μL of Tf (5 mg/mL) was added to 1184 μL of AuNPs and 384 μL of ddH_2_O. This ratio was the best one that showed no AuNPs spectral distortion after addition to the AuNPs (optimization data are not presented). The dispersion was incubated in a thermomixer (Eppendorf, Hamburg, Germany) at 1000 rpm and 4 °C overnight.^14,20^ The dispersion was then centrifuged at 15,000*g* for 30 min, and the supernatant was examined at the indicated excitation/emission wavelengths to determine the amounts of unbound Tf. The pellet was washed three times with RNase-free ddH_2_O and used for detection and cytotoxicity analyses.

### Conjugation of AuNPs to AntimiR-135b and AntimiR-Load Assessment

AntimiR-135b was designed during our previous research to specifically inhibit mammalian microRNA-135b. The RNA sequence used was 5′ rUrCrA rCrArU rArGrG rArArU rGrArA rArArG rCrCrA rUrA - 3′ labeled at 5′ with Alexa Fluor 488.^23^ The stock solution of 100 μmol of antimiR-135b was diluted to 0.03125 μmol, and then 1/2 serial dilutions were performed in a 96-well microtiter plate. The excitation/emission wavelengths were 488 and 520 nm, and the fluorescence intensity was determined in each well. The assay was performed three times, and the standard curve was generated. To conjugate the AuNPs with antimiR-135b, different amounts of AuNPs were conjugated with different amounts of antimiR-135b (50 μL of AuNPs + 50 μL of RNA (50 mmol), 60 μL of AuNPs + 40 μL of RNA (40 mmol), 70 μL of AuNPs + 30 μL of RNA (30 mmol), and 80 μL of AuNPs + 20 μL of RNA (20 mmol)). Finally, 118 μL of antimiR-135b at a concentration of 100 μmol was added to 1184 μL of the AuNPs and 282 μL of ddH_2_O dispersion. This amount was the best one that showed no AuNPs spectra distortion after addition to the AuNPs (optimization data are not presented). The dispersion was incubated overnight in a thermomixer at 1000 rpm at 4 °C.^14,20^ The dispersion was then centrifuged at 15,000*g* for 30 min. Because of the high fluorescence intensity of the conjugated fluorophore, the supernatant was first diluted and then examined at 488/520 nm wavelengths to determine the amounts of unbound antimiR-135b. The pellet was washed three times with RNase-free ddH_2_O and used for detection and cytotoxicity analyses.

### Conjugation of AuNPs to PTX and PTX-Load Assessment

The standard curve of PTX (1 mg/mL, Sigma-Aldrich, Prague, Czech Republic) stock solution was constructed by using 1/2 serial dilutions of the drug in a 96-well plate (the first well contained 1 mg/mL PTX). The absorbance of the different concentrations of the drug was determined by spectrophotometry at a wavelength of 230 nm.^26^ To conjugate the AuNPs with PTX, 160 μL of PTX (1 mg/mL) was added to 1184 μL of AuNPs and 240 μL of ddH_2_O and incubated overnight in a thermomixer at 1000 rpm at 4 °C. This amount was the best one that showed no AuNPs spectra distortion after addition to the AuNPs (optimization data are not presented). The dispersion was then centrifuged at 15,000*g* for 30 min, and the supernatant was examined at the same wavelength (λ_max_ 230 nm) to detect amounts of unbound PTX. The pellet was washed three times with RNase-free ddH_2_O and used for detection and cytotoxicity analyses.^19^

### Conjugation of AuNPs to PTX, AntimiR-135b, and Tf (FP Formation) and Load Assessments

After the standard curve was established and conjugation of each molecule with the AuNPs was demonstrated, PTX, antimiR-135b, and Tf were added to the AuNPs. For this purpose, 32 μL of Tf stock solution (5 mg/mL), 1184 μL of AuNPs, 118 μL of antimiR-135b (100 μmol), 160 μL of PTX stock solution (1 mg/mL), and 90 μL of ddH_2_O were combined and incubated overnight in a thermomixer at 1000 rpm at 4 °C. The dispersion was centrifuged at 15,000 g for 30 min, and the supernatant, after dilution, was analyzed for fluorescence intensity (for Tf and antimiR-135b) and optical density (for PTX) at the above wavelengths to estimate the amounts of individual unbound molecules in the supernatant and subsequently the amounts of molecules attached to the AuNPs surface. FP was washed three times with RNase-free ddH_2_O and used for detection and cytotoxicity analyses.

For the purpose of this study (complex FP effect), we used PTX in its sub-inhibitory concentration (IC50) concentration (determined by MTT assay, see below), so we could observe its effect on cell morphology and apoptosis (histology analysis) as well as study the accumulation and effect of inhibitory RNA (TEM and qPCR analyses).

### Proof of Conjugation

To demonstrate that the final AuNP conjugate consisted of AuNPs, Tf, PTX, and antimiR-135b, the FP samples were analyzed using spectrophotometry, zetasizer, and FTIR techniques, as mentioned earlier.

Data from the size and zeta potential analyses were compared using analysis of variance (ANOVA) in SPSS software as an online tool (available at https://astatsa.com/OneWay_Anova_with_TukeyHSD). p-values of ≤0.05 and ≤0.01 were considered as significant.

To confirm Tf conjugation to AuNPs, the conjugates (both AuNPs-Tf and FP) were analyzed by using a liquid chromatography–mass spectrometry system (LC–MS) (Agilent 1200 series, Agilent Technologies, USA) operated in positive data-dependent mode and connected to a timsToF Pro PASEF mass spectrometer with CaptiveSpray (Bruker Daltonics, USA). A C18 trap column (UHPLC fully porous polar C18 2.1 mm ID, Phenomenex) and a C18 column (Luna Omega 3 μm Polar C18 100 Å, 150 × 0.3 mm, Phenomenex) were used in this experiment. Results were analyzed using PEAKS Studio 10.0 software (Bioinformatics Solutions, Canada) and the UniProt database (all taxa, 11/2021).^27^

To confirm the conjugation of PTX to AuNPs, the supernatants of the conjugates (both AuNPs-PTX and FP) were analyzed using high-performance liquid chromatography (HPLC). For the analysis of PTX, the same HPLC instrument was used as previously published.^28^ Water-containing mobile phases were filtered through a 0.22 μm GS filter (Millipore, Billerica, MA, USA) and degassed in an ultrasonic bath for 10 min before use. The mobile phases consisted of 5% (v/v) methanol in water (phase A) and methanol (phase B). Both phases were acidified with 0.01% acetic acid. A Kinetex 5 μm C18 column (250 × 4.6 mm, Phenomenex) with a guard column was used for analysis. Gradient elution started at 30% B (marked as 0 min) and increased linearly to 100% B within 15 min at a flow rate of 1 mL/min. UV detection was performed at 227 nm. The amount of PTX was quantified by using a calibration curve. A standard solution (1 mg/mL PTX in DMSO) was further diluted in methanol to final concentrations of 20, 10, 2, 0.2, and 0.1 mg. mL^–1^. The calibration plot was constructed by plotting the integrated peak areas of PTX against the concentration. The following linear regression equation and correlation coefficient were obtained: y = 12574x–11,860, R^2^ = 0.9998.

To confirm the conjugation of antimiR-135b with AuNPs, the pellets of the conjugates (both AuNPs-antimiR-135b and FP) were analyzed by agarose gel electrophoresis. The migration delay of the conjugates compared with the control as a sign of successful conjugation was evaluated.^29,30^

To assess the stability of FP, as well as the release of PTX and antimiR-135b, FP (300 μL) was freeze-dried, diluted in 300 μL of phosphate buffered saline (PBS) at two different pH levels (pH 7.4 and pH 6.0), and incubated at 37 °C. At various time points (30 min, 1, 2, 5, 8, 24, and 48 h), the samples were centrifuged (15,000 g, 30 min), 20 μL of PBS was removed, and the concentrations of PTX and antimiR-135b were analyzed using a Tecan spectrophotometer. For PTX, the supernatant was analyzed for absorbance at 230 nm, and for antimiR-135b, the fluorescence intensity at 488 and 520 nm was measured. The analyzed samples were then returned to the rest of the samples, and the incubation was continued.^31^

### Cytotoxicity Assay (MTT Assay)

To analyze the activity of the conjugates in cell culture, an MTT assay was performed for FP and controls, including AuNPs and PTX alone. For this purpose, a 96-well tissue culture plate (JETBiofil, Guangzhou, China) was used, and 2 × 10^3^ cells/mL of a 4T1 cell line (cell line derived from mammary adenocarcinoma of BALB/c mouse strain, ATCC CRL −2539) were added to 4 rows of the plate. The other 4 rows were filled with the same amounts of NIH/3T3 (cell line of immortalized fibroblasts derived from NIH/Swiss mouse strain, ATCC CRL −1658) cells. The culture medium for the 4T1 cell line was Roswell Park Memorial Institute Medium 1640 (RPMI-1640, Sigma-Aldrich, Prague, Czech Republic) supplemented with 10% fetal bovine serum (FBS, Gibco, Massachusetts, USA), 44 μg/mL gentamicin (Sandoz, Novartis Company, Prague, Czech Republic), and 4.5 g/L glucose (Sigma-Aldrich, Prague, Czech Republic). The culture medium for NIH/3T3 cells was Dulbecco’s Modified Eagle Medium (DMEM, Sigma-Aldrich, Prague, Czech Republic) supplemented with 10% FBS and 50 mg/L gentamicin. After incubation of the plate at 37 °C and 5% CO_2_ overnight (reaching confluency 60%), cells in 4 rows of the plate were challenged with 1/2 concentration of the FP obtained by titration (the pellet of the conjugate was dissolved into 250 μL of appropriate media). Wells in rows 4 to 8 were loaded with PTX. For PTX as a control, a concentration equal to the determined amount of PTX in the FP was used. Six wells (3 wells for each cell line, D 10–12 and H 10–12) served as untreated controls and contained only the cells.

After overnight incubation, 100 μL of appropriate media with 20 μL of 5 mg/mL 3-(4,5-dimethylthiazol-2-yl)-2,5-diphenyltetrazolium bromide (MTT, EMD Millipore, CA, USA) in PBS were added into all experimental wells, and cells were incubated for another 4 h. Formazan crystals were dissolved in dimethyl sulfoxide (DMSO, Sigma-Aldrich, Prague, Czech Republic), and absorbance at 570 nm was analyzed in each well using a Tecan spectrophotometer at a reference wavelength of 630 nm. The cell survival rate (%) and therefore the percentage of half-maximal IC_50_ was calculated by this formula: cell survival rate (%) = (a–c)/(b–c) × 100 (a = absorbance of each well containing conjugate or drug, b = absorbance of the control well without any conjugate or drug, and c = absorbance of the blank).^32^

### In Vivo Experiment

The in vivo study was performed with respect to the Czech Law on Animal Protection Act no 246/1992 and was approved by the ethical committee of the Czech Academy of Sciences with the number 14–2022-P. Twenty Balb/c mice (female, 6 weeks old) were used in the study. The mice were randomly housed in cages with 5 animals/cage and received food and water *ad libitum*. To induce mammary tumors, a suspension of 10^6^ freshly prepared 4T1 cells in PBS was injected subcutaneously into the fourth left mammary fat pad. The mice were kept for tumor development for 14 days and observed every 2 days. After the developed tumor reached a diameter of approximately 5 mm, the treatment was started. Each cage was considered as a group and treated separately with PBS, AuNPs, PTX, and FP. The administration dose was 50 μL of sub-IC_50_ concentration of each sample (i.e., AuNPs, PTX, and FP) determined by MTT assay, and the injection was performed on three consecutive days into four regions of peripheral tumor tissue. Tumor size was measured before the start of treatment and after the last treatment. Under deep anesthesia (using ketamine/xylazine), one animal from each group was sacrificed 1 h after the third dose by cervical dislocation, and the tumor tissue was harvested.^23^ These animals are referred to here as group I. The remaining mice were sacrificed in the same manner 5 days after administration of the last dose. The diameter of each tumor outside the body was measured quickly, and the tumor volume was calculated according to the formula: (A × B × C)/2.^33^ These animals are referred to here as group II.

Each tumor sample was divided into three parts immediately after size measurement, one part was placed in the cold and fresh fixator and stored at 4 °C for TEM, the second part was placed in fresh formaldehyde 4% and stored at 4 °C for hematoxylin and eosin staining (H & E staining), and the third part was homogenized and the retrieved cells were kept in about 65% ethanol at −20 °C for GF-AAS and qPCR (described below).

### TEM and EDS Analysis

The samples from group I were cut into small cubes (1 mm^3^) and were fixed immediately in the fixator containing 3% formaldehyde and 1% glutaraldehyde in 0.1 M sodium cacodylate buffer (pH 7.4) and preserved at 4 °C. The next day, the sections were washed in sodium cacodylate buffer and post fixed with 1% OsO_4_ in sodium cacodylate buffer. After washing steps with the same buffer and water, different concentrations of acetone in water were used and embedded in Epon-Araldite resin. After polymerization of the resin, the sections were prepared using a microtome. Jeol JEM-F200 TEM operated at 200 kV and equipped with a TVIPS XF 416 CMOS camera was used for imaging. JED 2300 X-ray spectrometer was applied for obtaining EDS spectra from the specific detected AuNPs, and the raw spectra were analyzed using the standardless zeta factor method in Jeol Analysis Station software.

### H & E Staining

The tumor samples from group II were fixed and embedded in paraffin blocks. Using a microtome, sample slices of 5 μm thickness were prepared and stained by the H & E method.^34^ Differences in parameters such as neoplastic, necrotic, apoptotic, and inflammatory cells as well as the amount of blood accumulation were analyzed and compared in the different groups under a light microscope.

### Homogenization of the Tumor Samples and Internalization of AuNPs Analysis

To separate the cells, tumors from group II were homogenized in PBS, centrifuged at 1500 g for 5 min, and resuspended in PBS/70% ethanol (1:9). In this stage, cells were stored at −20 °C until further processing. To evaluate cellular internalization of FP and AuNP samples, 1 mL of each sample was analyzed with GF-AAS, and the amounts of Au present in the tumor-derived cells were calculated.

### qPCR

qPCR analysis was employed in order to assess the inhibitory effect of antimiR-135b on its target (i.e., miR-135b) in the FP group. The control samples (i.e., AuNPs, PTX, and PBS groups) were analyzed as well and used as controls. All samples were from group II. The microRNA was isolated using High Pure miRNA Isolation Kit (Roche, Prague, Czech Republic),^23^ and its amount was determined using Invitrogen Qubit RNA High Sensitivity Kit (Thermo Fisher Scientific, Prague, Czech Republic). High-Capacity cDNA Reverse Transcription Kit (Thermo Fisher Scientific, Prague, Czech Republic) was applied for cDNA synthesis. The miR-specific reverse transcription primers were miR-16 (assay 000391, Thermo Fisher Scientific, Prague, Czech Republic) and miR-135b (assay 002261). miR-specific RT-PCR primers and TaqMan Universal PCR Master Mix (No AmpErase) were used for amplification, and each sample was tested in triplicate. The amplification and data analysis employed an iQ5 Real Time PCR Detection System (BioRad, Prague, Czech Republic) and an iQ5 Optical System Software 2.1 (BioRad, Prague, Czech Republic), respectively. The miR-135b expression was normalized to the miR-16 internal control.^23^

## Results and Discussion

### Cultivation of Fungi and Production of AuNPs

**Figure 1:**
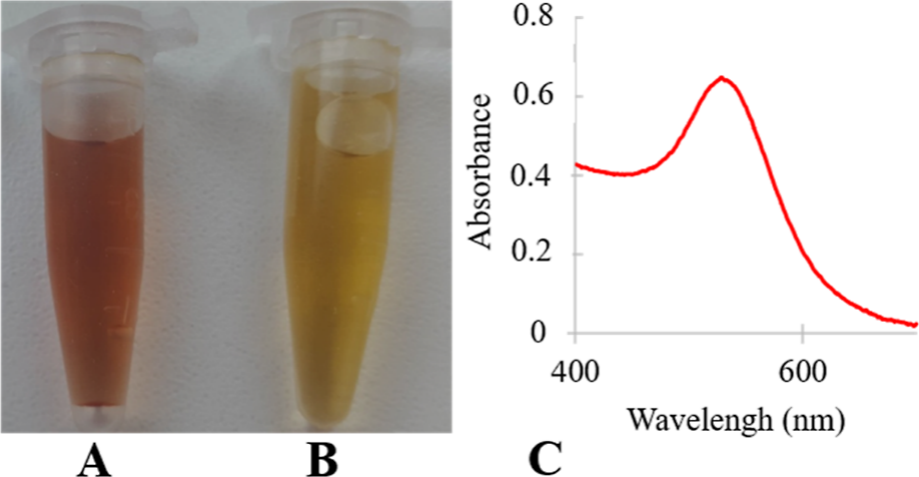
Preparation of AuNPs by challenging the supernatant from a microbial culture with Au^+^ ions. (A) Color-changed supernatant after formation of AuNPs, (B) control supernatant, and (C) spectrophotometry of the AuNPs dispersion showing the maximum absorption peak at 528 nm.

The results showed that the color of the supernatant containing HAuCl_4_.3H_2_O changed from yellow to crimson after incubation, indicating the formation of AuNPs (Figure 1A). Color of the nonchallenged control flask remained unchanged (Figure 1 B).

### Characterization of the AuNPs

#### Spectrophotometry

Spectrophotometry showed that AuNPs exhibited a maximum absorption peak at 528 nm, confirming the presence of AuNPs in the dispersion (Figure 1C).

### TEM and EDS

**Figure 2:**
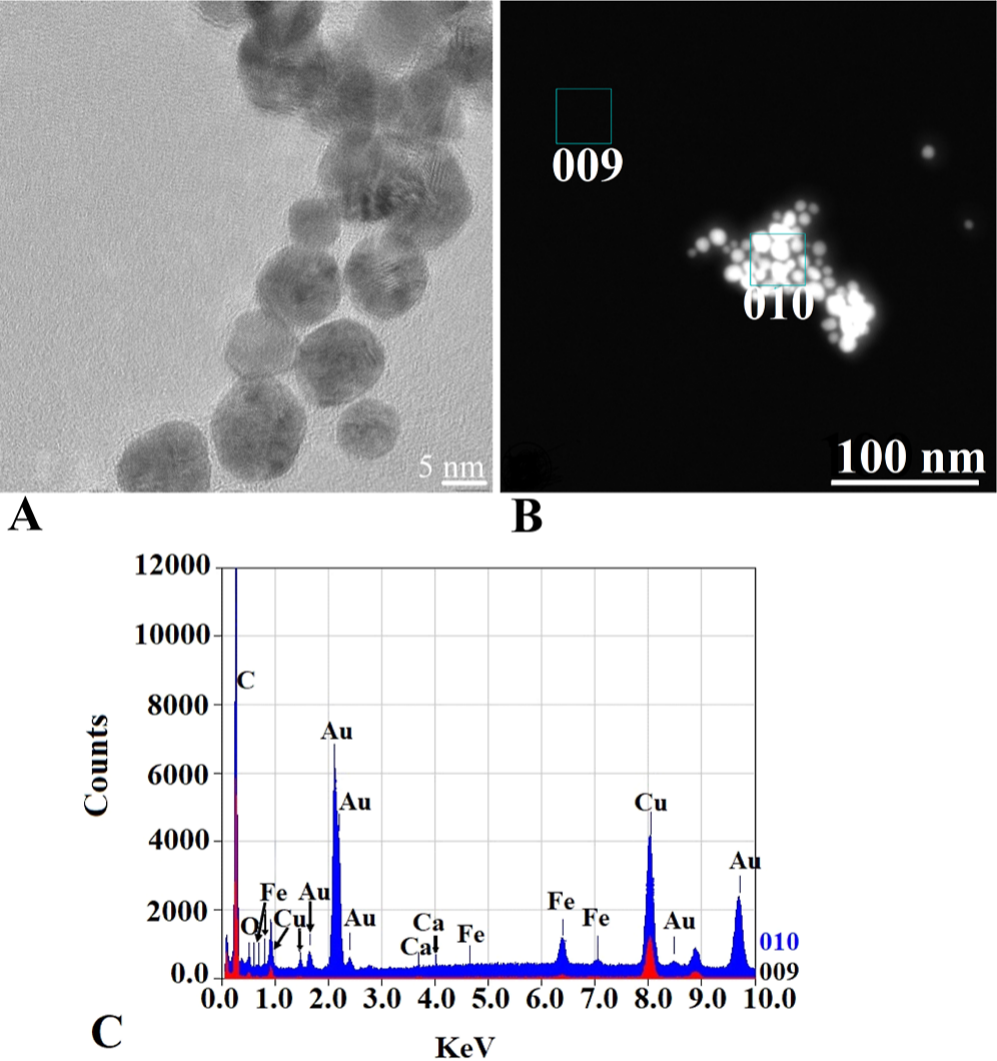
Representative TEM and EDS analyses of the biologically produced AuNPs. (A) TEM (scale bar = 50 nm) and (B) location of areas that were analyzed by EDS (scale bars = 5 and 100 nm), the spot 009 represents the background, and the spot 010 represents the AuNPs rich area. (C) EDS analysis of AuNPs (in blue) and background (in red).

The results from TEM confirm the presence of AuNPs in the samples with mostly polygonal shapes and an average size of 15 nm. EDS confirmed the presence of elemental Au in the sample. Figure 2 shows the obtained results.

The Au peaks in Figure 2C confirm the presence of elemental Au in the sample. There are several other peaks, such as Cu peaks belonging to the copper grid and C peak, which confirms the presence of organic matter on the AuNPs surface.

### Zetasizer Analysis

The hydrodynamic diameters (sizes estimated by DLS) and surface zeta potentials of the AuNPs as well as subsequent conjugates are listed in Table 1. The average hydrodynamic diameter of the original AuNPs was about 13 ± 1 nm, and they possessed a negative zeta potential of about −35 mV (Table 1).

**Table 1:** Statistical Analyses of Differences in Hydrodynamic Diameter (Size) and Zeta Potential (Charge) of Default AuNPs and AuNPs Conjugated with Different Cargoes^a^

charge size	AuNPs	AuNPs-Tf	AuNPs-PTX	AuNPs-antimiR	FP
AuNPs	N/A	0.89	0.001**	0.08	0.02*
AuNPs-Tf	0.001**	N/A	0.002**	0.03*	0.052
AuNPs-PTX	0.001**	0.89	N/A	0.001**	0.56
AuNPs-antimiR	0.02*	0.001**	0.001**	N/A	0.001**
FP	0.001**	0.27	0.18	0.003**	N/A

a *P*-values are based on five individual measurements, detailed in Table S1. Significant *p*-values are in bold, the *p*-values ≤0.01 are marked with **, and the *p*-values ≤0.05 are marked with *.

### GF-AAS

The GF-AAS showed that a total of 2.24 ± 0.10 mg of Au was found in the 1184 μL of AuNPs in the ddH_2_O sample.

### Conjugation and Loading Efficiency Tests

As shown in Figure S1A, the color of the dispersions was constant after conjugation of the AuNPs with individual components, PTX, antimiR-135b, and Tf as well as with their mixture (FP). The conjugates were resuspended in sera-supplemented culture media (DMEM with 10% FBS and gentamycin), proving that the AuNP conjugates remain stable and the presence of sera and the incubation of AuNPs with the cargoes did not affect the nature of the AuNPs in respect to agglomeration.

### Determination of the Loading Amounts of Each Molecule in AuNPs-Tf, AuNPs-antimiR-135b, AuNPs-PTX, and the Prepared FP

Standard curves of Tf, antimiR-135b, and PTX were determined (Figure S1B, C) in order to estimate the load of the individual components within AuNP conjugates. The estimated amount of each molecule in AuNPs-conjugate was determined using an equation based on the fluorescence intensity of the supernatants (in the case of Tf and antimiR-135b) or absorbance at 230 nm of the supernatant containing PTX.

Considering the standard curves, we estimated that on average AuNP conjugates contained 0.041 mg/mL of Tf (in the AuNPs-Tf conjugate) or 0.66 μmol of antimiR-135b (in the AuNPs-antimiR-135b conjugate) per 1 mg of the present AuNPs. In the case of the AuNPs-PTX conjugate as well as the FP all (0.16 mg), the added PTX was present within the conjugate (containing on average 2.24 mg of AuNPs). Next to PTX, the FP consisted of 0.83 μmol of antimiR-135b and 0.037 mg/mL of Tf per 1 mg of AuNPs.

### Proof of Conjugation

#### Spectrophotometry

**Figure 3:**
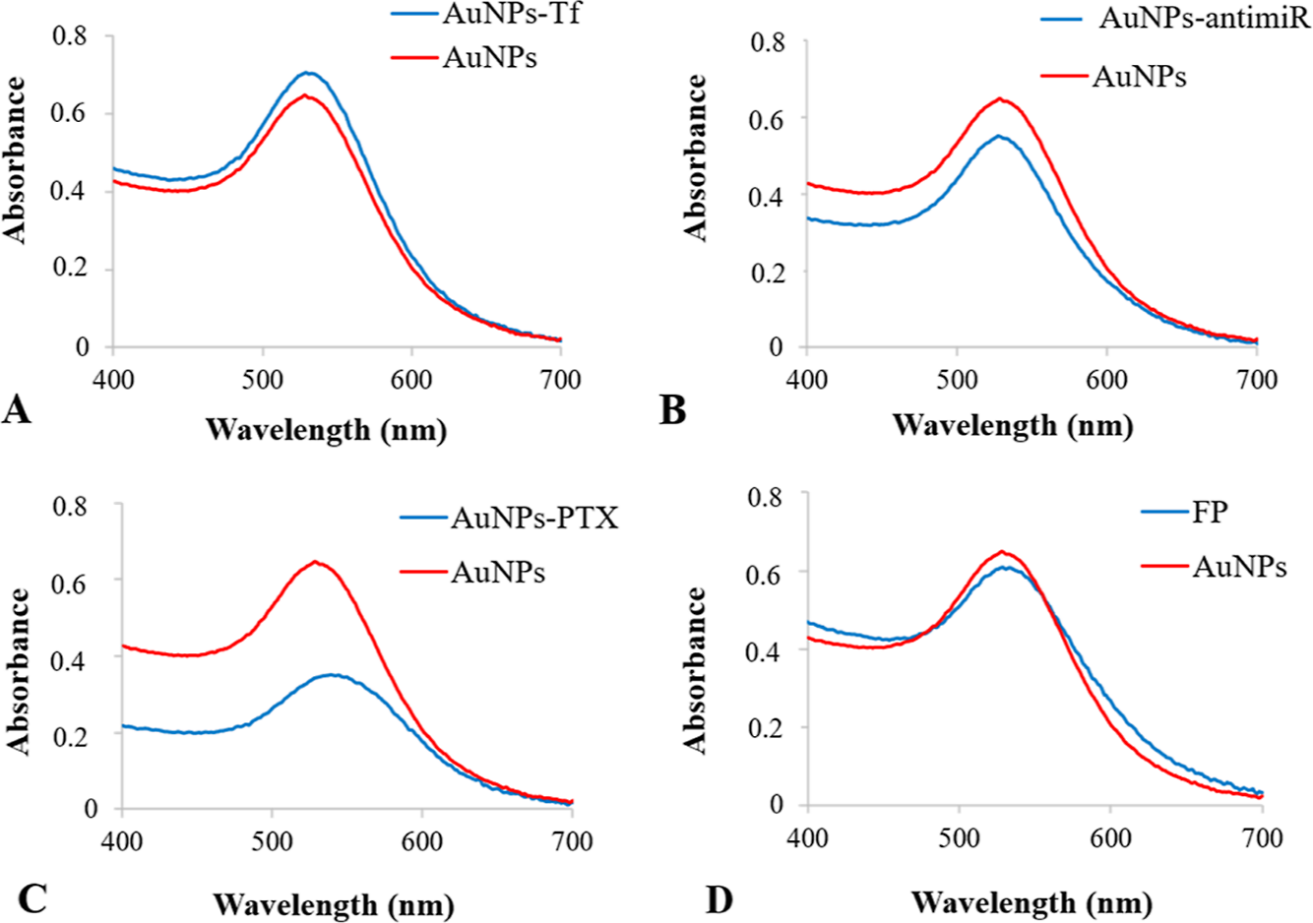
Representative spectral distribution exhibited by individual AuNP conjugates (blue) and their comparison to default AuNPs (red). (A) AuNPs-Tf, (B) AuNPs-antimiR, (C) AuNPs-PTX, and (D) FP.

The maximum absorbance peak of each conjugate compared to original AuNPs as control was determined using NanoDrop, and representative spectra are shown in Figure 3.

The maximum absorption peak of default AuNPs (528 nm) shifted based on conjugation with a particular molecule: AuNPs-Tf (529 nm), AuNPs-antimiR-135b (527 nm), AuNPs-PTX (539 nm), and FP (529 nm). The shift in the maximum absorption peak points to successful conjugation.

The FP remained stable, and as shown in Figure 3D, the slope of the peak was well maintained. Although the spectral slope of the AuNPs-PTX was blunt compared to the default AuNPs, the FP samples exhibited a sharp slope of the spectrum, indicating that the AuNPs were uniform in size.

Stability of the AuNPs after conjugation with the cargoes can be determined by observing slope changes as we previously reported with respect to nucleic acids-AuNPs conjugates.^35^ Based on that experience, we concluded that in the current study the AuNPs were stable after conjugation with different molecules and after preparation of the FP.

#### Hydrodynamic Diameter and Zeta Potential of the Conjugates

Hydrodynamic diameter shows the size of the nanoparticle core together with the surface molecules. Such a size evaluation might differ from estimates based solely on the AuNP core. After the conjugation of different cargoes with AuNPs, the AuNPs exhibited a size below 100 nm. All conjugates also carried remarkable negative zeta potentials, supporting our previous statements regarding the retained stability of AuNPs after any conjugation (Table 1). The particle size distribution histogram showing results for the AuNPs before and after conjugation with the three different molecules is shown in Figure S3.

Significant differences in size and surface charge were evaluated by ANOVA analysis and the posthoc Tukey HSD test.

As expected, all conjugates were significantly larger (showed a larger hydrodynamic diameter) in comparison to the default AuNPs. Furthermore, there were no significant differences in size among AuNPs-Tf, AuNPs-PTX, and FP conjugates, but all these conjugates were significantly larger than AuNPs-antimiR.

Regarding the surface charge, we found no significant difference between default AuNPs and AuNPs-Tf or AuNPs-antimiR conjugates. On the other hand, conjugation of PTX resulted in a significant change of zeta potential in comparison to other conjugates as well as default AuNPs. While there was no difference in surface charge between AuNPs-PTX and FP, we speculate that the PTX component plays a key role in determining the final surface charge of the FP.

In our previous studies, we investigated the relationship between the zeta potential and the stability of AuNPs. The AuNPs produced had a high negative zeta potential initially, but after incubation with cargoes, there were usually some changes in these amounts. Stable nanoparticles typically maintain the same or even more negative charge, while unstable ones increase their charge.^15,35^ It seems that in the current study, the addition of PTX caused a charge increase. However, it was not sufficient to destabilize the AuNPs conjugate (see Table S1 for more information).

#### Proof of the Cargo Conjugation to AuNPs by FTIR

**Figure 4:**
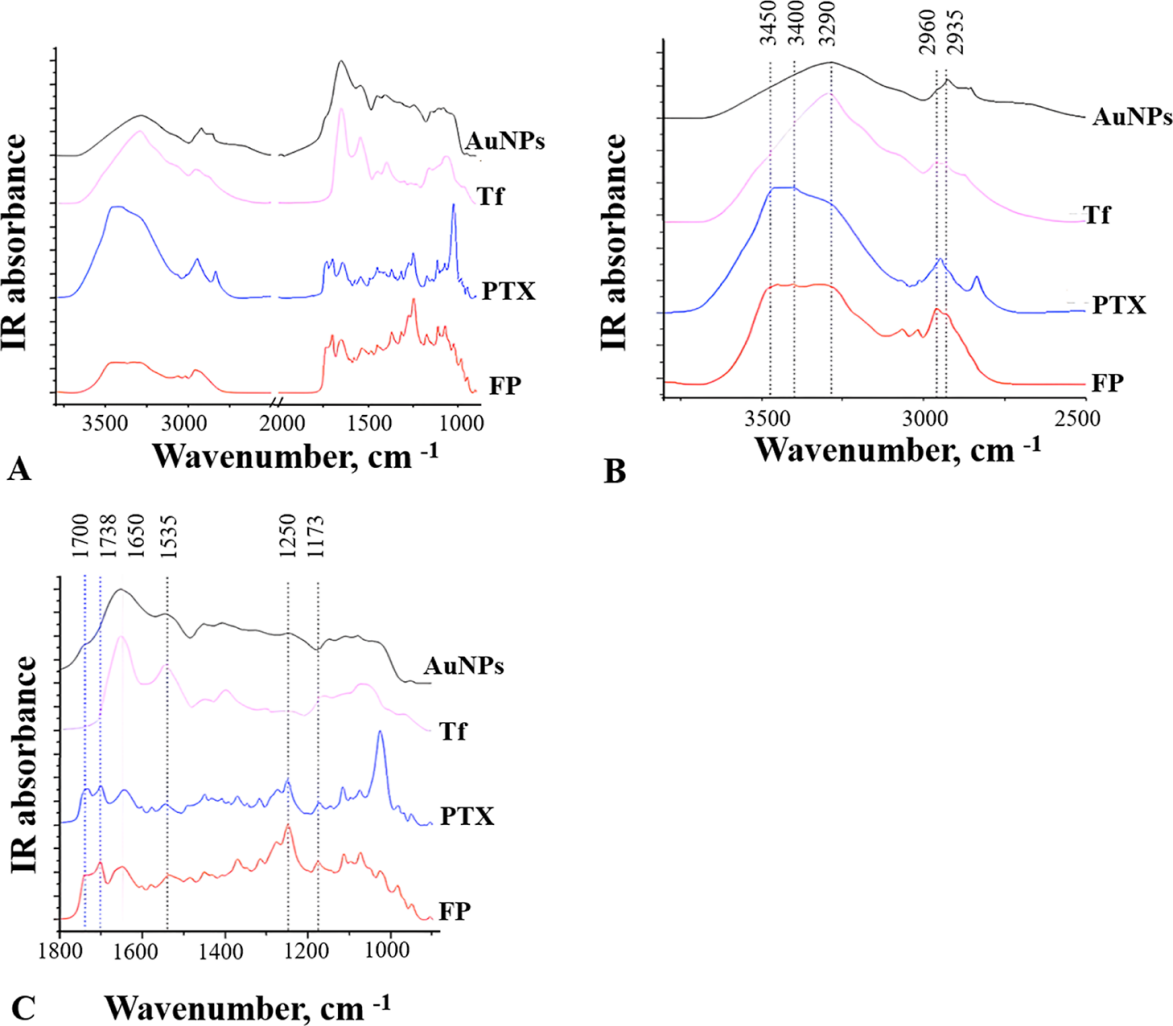
FTIR spectra of FP compared to control spectra of the individual components AuNP, PTX, and Tf. (A) Full-range IR spectra from 3500 to 900 cm^–1^, (B) detailed IR spectra from 3500 to 2500 cm^–1^, and (C) detailed IR spectra from 1800 to 900 cm^–1^.

The FTIR spectra of FP and the individual components, i.e., AuNPs, Tf, and PTX were analyzed. Representative spectra of the FP conjugate compared to the individual components as controls are shown in Figure 4.

As presented in Figure 4A, B, the FP spectrum has overlapped multiple peaks of N–H and O–H stretching vibration spreading from about 3480 to 3280 cm^–1^, while the AuNP as well as Tf have a broad peak centered at 3290 cm^–1^ and PTX has a broad peak centered at about 3450 to 3400 cm^–1^. The overlapped peak of FP likely sums signals originated from PTX with AuNP and/or Tf. Similar to Tf spectra, the C–H vibration region of FP exhibits two peaks at 2960 and 2935 cm^–1^, which corresponds to - C(sp^3^)-H vibrations of CH2/CH3 groups present in protein side chains, and two peaks of = C(sp^2^)-H stretching vibrations, which are originated from PTX. Further, the peak at about 1450 cm^–1^ represents the CH_2_ scissoring mode, and it is present in each compound (Figure 4C).

In the fingerprint IR absorption region (Figure 4C) of the FP spectrum, majority of peaks possess the same positions as the PTX spectrum, clearly indicating the presence of PTX. The PTX strong sharp peaks at 1733 and 1701 cm^–1^ are shifted in the FP spectrum toward 1733 and 1702 cm^–1^ and correspond to C=O stretching and C=C stretching, which is also supported with peaks of C=C bending of PTX at 905 and 983 cm^–1^ for both compounds. As all tested components contain amides, they all have amide I and amide II peaks at about 1650 and 1540 cm^–1^, and so does the FP. There are also common peaks at 1250 cm^–1^ denoting C–N stretching and at 1071 to 1081 cm^–1^ representing absorption of C–O stretching.

There are two unique weak peaks at the FP spectrum that are not present in other samples (at 1017 cm^–1^ (shoulder) and 1017 cm^–1^), which may correspond to C–O ribose stretching of nucleic acid. Major IR bands of nucleic acids are at about 1247 and 1085 cm^–1^ (-PO4 stretching) and about 1655 or 1700 cm^–1^ (C=O stretching) that are present in PTX spectra and may be overlapped (Figure 4C). We suggest there is a small amount of nucleic acids within the FP, but it is arguable since the statement is based only on FTIR data.

#### Transferrin Assessment by LC–MS

Mass spectrometry analysis of AuNPs-Tf and the FP confirmed the presence of Tf in both samples, and the results are consistent with the FTIR results. Table 2 presents the adequate output data.

**Table 2:** LC–MS Output Set, Regarding the Presence of Tf on the Surface of AuNPs, from Both AuNPs-Tf and FP Samples

protein group	protein ID	accession	coverage (%)	intensity	avg. mass	description
AuNPs-Tf						
1	131,110	P02787|TRFE_HUMAN	77	1.08 × 10^6^	77,064	Serotransferrin OS = Homo sapiens OX = 9606 GN = TF PE = 1 SV = 3
**FP**						
1	131,110	P02787|TRFE_HUMAN	76	6.62 × 10^4^	77,064	Serotransferrin OS = Homo sapiens OX = 9606 GN = TF PE = 1 SV = 3
2	131,111	A5A6I6|TRFE_PANTR	70	4.14 × 10^2^	77,064	Serotransferrin OS = Pan troglodytes OX = 9598 GN = TF PE = 2 SV = 1

### Paclitaxel Assessment by HPLC

**Figure 5:**
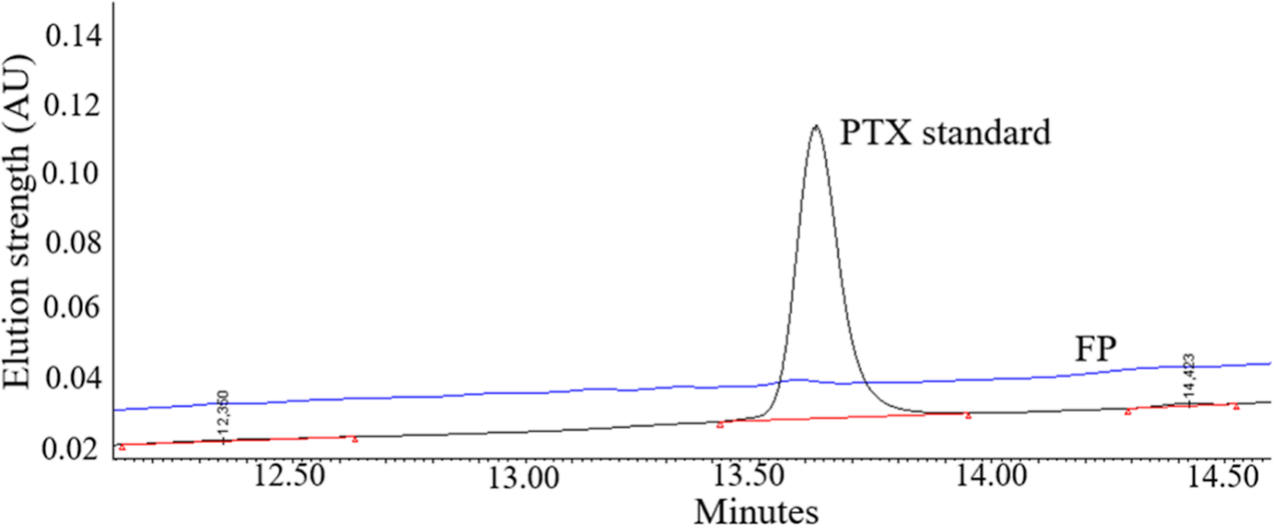
Determination of free PTX remaining in the supernatant after preparation of FP (starting concentration of PTX 107 μg/mL, blue line). Chromatogram of standard solution of PTX (20 μg/mL, black line) is given for comparison.

HPLC analysis (Figure 5) confirmed the effective binding of PTX within the FP and AuNPs-PTX conjugates, and the obtained data agree with the FTIR analysis. There was no free PTX detected in supernatants originating from the FP and AuNPs-PTX samples. Representative analysis of the FP supernatant is shown in Figure 5. Full chromatograms are available in Figure S2A. The estimated concentration of PTX in the tested conjugate was about 107 μg/mL. Increasing the PTX concentration to 545 μg/mL led to saturation of PTX within the AuNPs-PTX conjugate, and traceable amount of PTX could be detected in the supernatant (Figure S2B).

Based on the HPLC results, we conclude that all of the PTX (in the concentration used in FP) was fully associated with the AuNPs. Applying approximately five times higher concentration of PTX saturated the AuNPs surface.

### RNA Assessment by Electrophoresis

**Figure 6:**
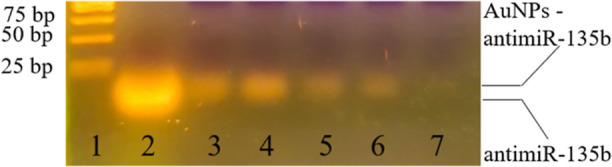
Confirmation of the antimiR-135b-AuNPs conjugation. Different concentrations of antimiR-135b are shown: (1) MassRuler low-range DNA ladder, (2) control antimiR-135b (100 mM), (3) AuNPs-50 mM of antimiR-135b, (4) AuNPs-40 mM of antimiR-135b, (5) AuNPs-30 mM of antimiR-135b, (6) AuNPs-20 mM of antimiR-135b, and (7) FP.

For final confirmation of the conjugation of different concentrations of antimiR-135b with AuNPs, the pellets of the conjugates were analyzed by agarose gel electrophoresis, which all showed oligonucleotide presence and a migration delay of the conjugates compared to the free control antimiR-135b (Figure 6).

RNA electrophoresis showed a detectable oligonucleotide (antimiR-135b) band in AuNPs conjugates as well as a visible migration delay of the antimiR when conjugated with AuNPs. These data are in agreement with our previous study^35^ where we reported that AuNPs have the ability to directly conjugate with different types of nucleic acids, including short single-stranded RNAs such as antimiR-135b.

**Figure 7:**
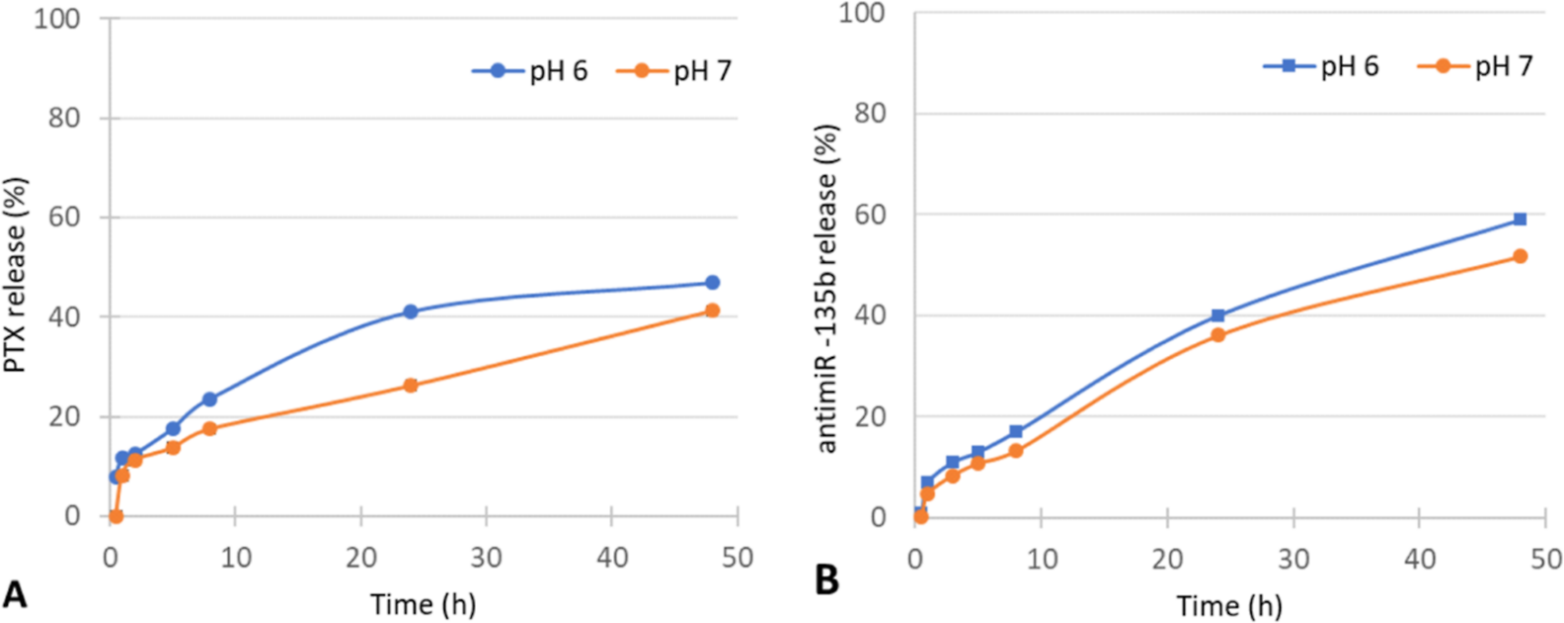
Release dynamics of (A) PTX and (B) antimiR-135b in two different pH levels and several time intervals. The release is shown as a percentage of control (starting amount loaded onto a nanoparticle).

To check the stability of FP and release of PTX and antimiR-135b, the FP was analyzed in two different pHs (pH 7.4 and pH 6.0) and in seven time intervals (i.e., 0, 30 min, 1, 5, 8, 24, and 50 h). The analyzed PTX and antimiR-135b concentrations are shown in Figure 7.

According to the data obtained from this study, the release of the molecules is faster in pH 6 compared to pH 7, with approximately 50% of both molecules remaining attached to the FP after 48 h of incubation. It is demonstrated that the release of both molecules from the AuNPs is faster at pH 6. It supports our hypothesis that at minimum, the RNA cargo releases from the nanoparticle under a low pH during maturation of endosomes into lysosomes. The FP uptake by endocytosis and accumulation within the endolysosome can be found below in Figure 10, and the inhibitory RNA effect can be found below in Figure 12.

In order to detect PTX release, we employed a dialysis bag first. However, this approach cannot be used in combination with AuNPs because they clog the pores of the bag. Additionally, the ratio of FP/PBS was 1/100 and detecting PTX and antimiR-135b at such low concentrations was challenging. On the other hand, the remnants of the cargo on the FP surface could not be accurately determined either because the absorbance and fluorescence spectra suffered from AuNP-mediated background noise.

England et al. have demonstrated that the incorporation of PTX inside two- and three-layered nonbiologically produced AuNPs results in differences in the loading and drug release efficiency of PTX. For instance, approximately 68.4% of PTX was loaded onto the surface of the two-layered AuNPs, while nearly 99.4% was loaded onto the surface of the three-layered AuNPs. During the initial 24 h of incubation with 1X PBS (pH 7.4) for the drug release study, approximately 3.8% of PTX was released from the two-layered AuNPs and 7.5% was released from the three-layered AuNPs.^36^ In comparison, our study revealed that 100% of the PTX was conjugated on the surface of the biologically produced AuNPs, and after 24 h incubation with 1X PBS (pH 7.4), over 20% of the PTX was released from the surface of the AuNPs. In a study by Manivasagan et al., chitosan-produced AuNPs were loaded with PTX and the amount of drug release in various pH values (5.5, 6.8, and 7.4) was investigated. They found that the highest release of PTX occurred in acidic pH, with approximately 40% of the drug being released at pH 7.4 and over 70% of the drug released at pH 5.5 after 24 h incubation.^37^ That study supports our findings regarding better drug release at acidic pHs. We suggest that variation in the PTX release from AuNPs is based on the nanoparticle surface and structure.

### Cytotoxicity Assay

**Figure 8:**
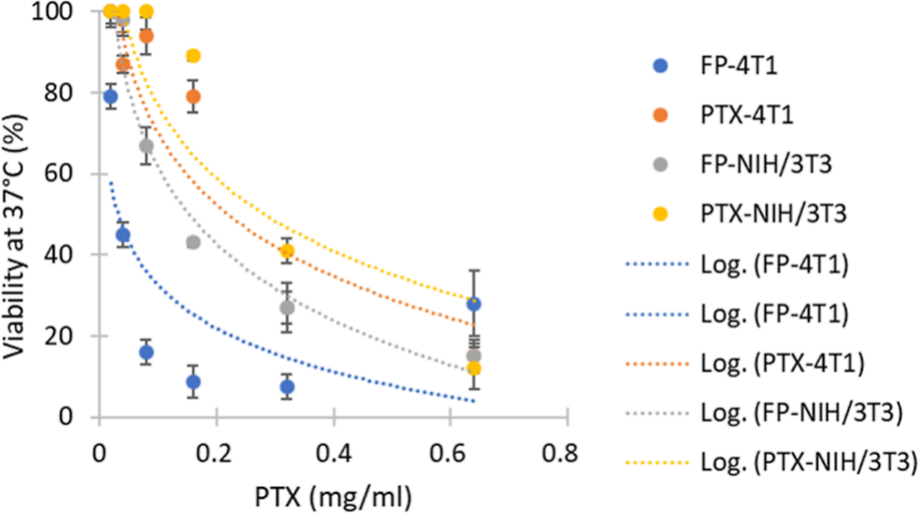
Logarithmic trendline presenting PTX-mediated toxicity with respect to its free form (PTX) or conjugation with the AuNPs within the FP. Viability (% of nontreated control) for cancer cell line 4T1 and noncancerous fibroblasts NIH/3T3 is shown. Cells were incubated at 37 °C for 24 h.

PTX is a component of the FP that is known for its cytotoxic effect on cancer cells. To evaluate PTX-mediated toxicity of the conjugates in cancer cells and noncancerous fibroblasts, we used equal amount of free PTX that was detected within the FP. We found that although both cell lines were inhibited by the FP, it was more notable in the 4T1 cancer cells than in the NIH/3T3 fibroblasts cells. The sensitivity of both cell lines to free control PTX was the same, but the sensitivity of the cancer cells to the PTX within the FP was higher than in the case of fibroblast. We conclude that the FP was more effective in interacting with, internalizing, and transfecting the 4T1 cancer cells because of its tumor-targeting properties (e.g., transferrin ligand). Figure 8 shows the calculated viability for the FP and PTX treatments in each cell line (averages of nine replicates). The calculated averages of replicates (including appropriate IC_50_) obtained from the difference between absorbance at 570 nm compared to the reference wavenumber (i.e., 630 nm) are listed in Table S2.

Overall, the results confirmed the higher inhibitory effects of FP against 4T1 cells compared to those of the other cell line (see Table S2).

### Evaluation of FP Conjugate In Vivo

**Figure 9:**
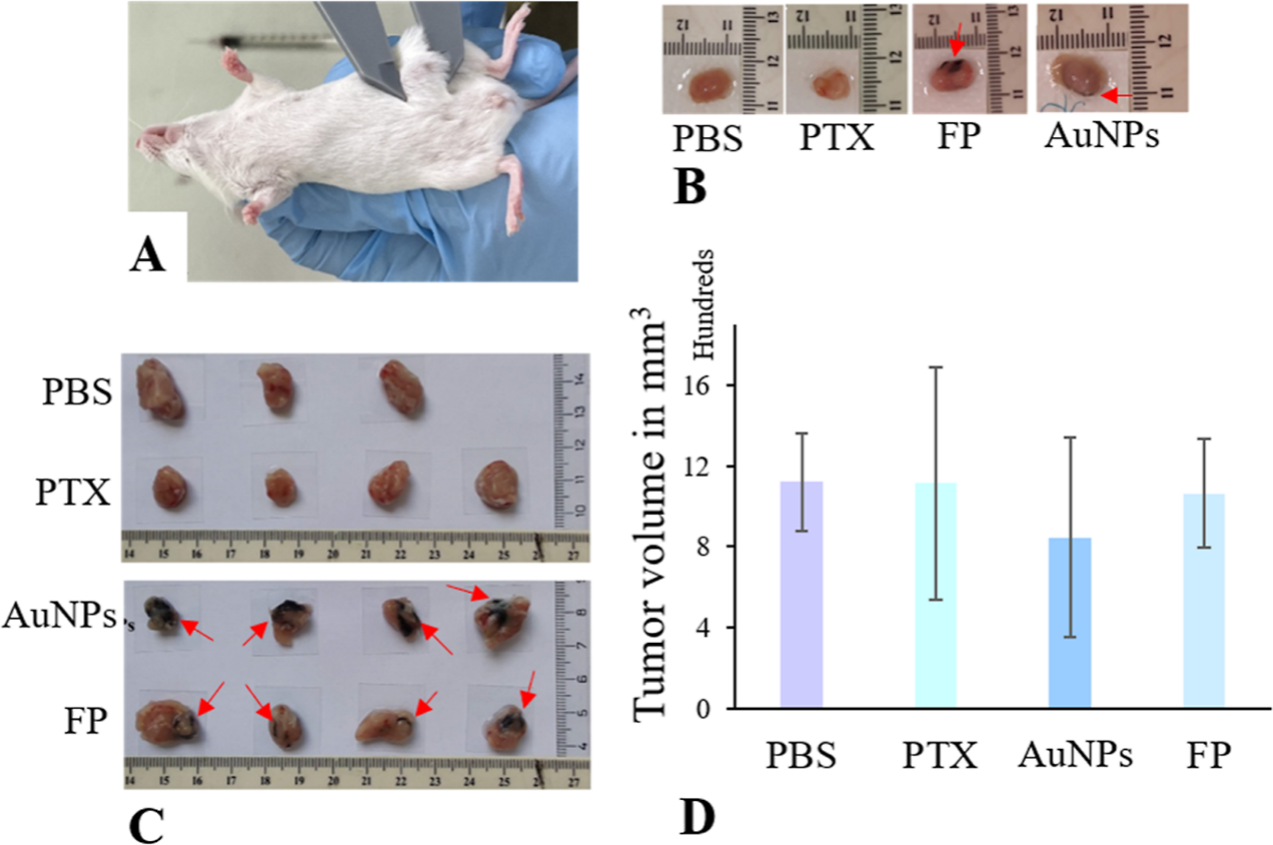
Tumor growth evaluation in vivo. (A) Developed BC in Balb/c mice and (B) tumors excised from experimental group I. (C) Tumors excised from experimental group II and (D) comparison of estimated tumor volume in animals from group II. Red arrows indicate AuNPs.

In order to test the applicability of the FP conjugate in vivo, we developed breast tumors in a Balb/c mouse model (Figure 9 A). Once the tumors reached about 0.5 cm, we performed treatment with FP, as well as AuNPs and PTX alone, and PBS. The groups treated with AuNPs and PTX alone served as controls for the effect judgment of the individual components. The group that obtained PBS represented nontreated control. All groups received treatment or PBS at the same time in three consecutive doses. One hour after administration of the last dose, one animal from each group was sacrificed (here referred to as group I). Five days after administration of the last dose, the remaining animals were sacrificed (here referred to as group II). Tumors obtained from sacrificed animals were measured and further analyzed. Figure 9B shows tumors excised from group I. In general, these tumors were smaller than those obtained from group II (in group II, the tumors could continue growing). Tumor samples obtained from group I were used solely to check the successful application of nanoparticles and their immediate uptake by tumor tissue. The application sites in the case of AuNPs and FP can be detected visually since the nanoparticles retain their crimson/violet color even after in vivo application (Figure 9B). To confirm prompt AuNP infiltration into tumor tissue, TEM analysis was used.

Tumor samples of group II underwent the same visual check of the AuNPs application (Figure 9C). The AuNPs were still well visible in tumors that received AuNPs alone as well as FP and the colorful spots especially in AuNPs parallel were larger in comparison to samples from group I. This may point to further penetration of the AuNPs into tumor tissue. Tumors excised from experimental group II were compared regarding size (Figure 9D). There are three tumors in the PBS group in contrast to the other groups because one animal died before the end of the experiment.

As presented in Figure 9D, tumor tissue volume (from group II) was not significantly reduced after treatment with FP, AuNPs, or PTX, when compared with the PBS group. The ANOVA with posthoc Tukey HSD test showed that there is no significant difference between the test groups (in all groups *p*-value > 0.05). PTX is a cytostatic drug that leads to tumor death and thus volume reduction. However, in our setup, we used a sub IC_50_ concentration of the drug to avoid the rapid death of tumor cells. That way we could learn about the FP internalization and its subsequent effect on surviving tumor cells. In the FP samples from experimental group II, we performed H & E staining to evaluate tumor structure and cell type distribution and qPCR to evaluate inhibition of target microRNA-135b in cancer cell cytoplasm.

### AuNPs Infiltration into Tumor Tissue In Vivo Confirmed by TEM and EDS Analyses

Group I tumor tissue was analyzed using TEM. Although mice were sacrificed 1 h after administration of the last dose (group I) to evaluate early infiltration of AuNPs into tumor mass and the likelihood of AuNPs agglomeration. In both experimental groups (I and II), the nanoparticles accumulated in several spots on the outside of the tumor (Figure 9B and C) and thus their agglomeration could occur.

**Figure 10:**
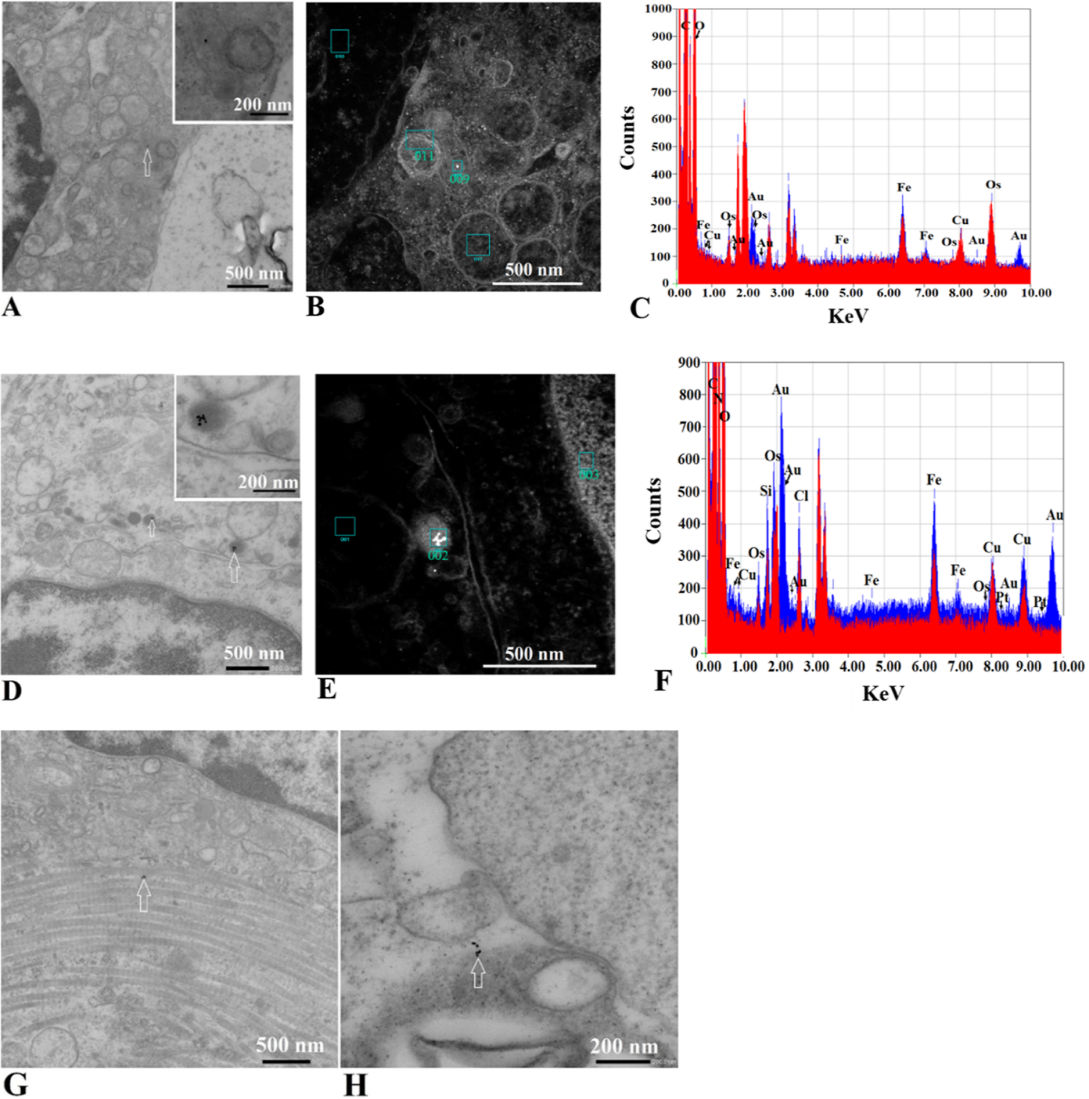
TEM and EDS analyses showing penetration of AuNPs alone and FP into tumor tissue 1 h after completion of treatment (experimental group I). (A) Distinguishable AuNPs from the AuNP parallel were seen individually and within the mitochondrion (white arrow, scale bar = 500 nm and insert = 200 nm). (B) Selected areas for EDS analysis (scale bar = 500 nm). (C) EDS analysis of areas 009 (blue) and 011 (red) from part B. (D) Distinguishable AuNPs from the FP parallel were seen individually or in clusters within the endolysosome (white arrows show AuNPs inside the electron-dense lysosome, scale bar = 500 nm and insert = 200 nm). (E) Selected areas for EDS analysis (scale bar = 500 nm). (F) EDS analysis of areas 002 (blue) and 003 (red) from part E. (G) AuNPs found within ER in AuNP parallel (white arrow, scale bar = 500 nm) and (H) clusters but not agglomerated AuNPs seen freely in the cytoplasm of the FP parallel (white arrow, scale bar = 200 nm).

The AuNPs were able to enter the cells in both treatment groups (AuNP alone and FP) and were seen in the cytoplasm, endosome, late endosome, or lysosome, and even in the mitochondria and endoplasmic reticulum (ER). Representative images are shown in Figure 10. To prove that the particles seen were AuNPs, a simultaneous EDS analysis was performed, and the Au content was confirmed (Figure 10C and F). The AuNPs were observed singly or in clusters in both treatment groups (AuNPs and FP). We found potential agglomeration in the endolysosome, vesicles possessing a low pH. This observation confirmed our previous report regarding the agglomeration of AuNPs in low pH.^17^ Since at the same time the AuNPs were also seen singly in the endolysosome, further analysis is necessary to elucidate that observation (e.g., by assessing the presence of AuNPs in the extracellular vesicles, see preprint: 10.26434/chemrxiv-2024–75npw). We hypothesize that releasing the cargo from the AuNP core in FP samples in response to low pH results in exposing the capping agent. Due to low pH, that capping agent (as well as the one in AuNP samples) undergoes molecule reorganization, shifting surface charge, and partial or complete aggregation.

There are some reports about the internalization method of nonbiologically produced AuNPs. For example, using endocytosis inhibitors and TEM analysis, it was revealed that uptake of the 20 nm AuNPs depended on clathrin-mediated endocytosis.^38^ The size, surface coating, and charge of the AuNPs are important in their uptake, and thus we plan to investigate the entry and intracellular fate of the biologically produced AuNPs in the near future.

Finally, after more than 2000 cell nuclei were examined by TEM, no AuNPs were detected in the cell nuclei. This suggests that AuNPs from both treatment groups (i.e., FP and AuNP parallels) penetrated the plasma membrane but not the nuclear membrane. This observation suggests that the biological AuNPs used in this report likely do not cause genotoxicity. Further analysis is required to confirm this result.

### Histology Analysis of the Tumor Tissues Evaluated by H & E Staining

Tumor growth depends on the balance between cell proliferation and cell death^39^ and particularly cells undergoing programmed cell death (i.e., apoptosis, necroptosis, ferroptosis, entosis, and pyroptosis)^40^ remain in the tumor tissue until their disintegration. Tumor-driven deregulation of apoptosis, which is the most studied programmed cell death, promotes cancer metastasis.^41,42^ PTX, that we used in our study, induces apoptotic cell death in tumor cells and stops the cell cycle by binding to microtubules.^43^ To evaluate the effect of the FP with respect to induction of cell death including apoptosis, we used sub IC_50_ doses of PTX in our setup. The applied PTX dose does not trigger excessive necrosis of the tumor tissue, as confirmed by minimal changes in tumor size (Figure 9D). The sub IC_50_ dose was effective though to induce apoptosis in cancer cells as revealed by H & E staining.

**Figure 11:**
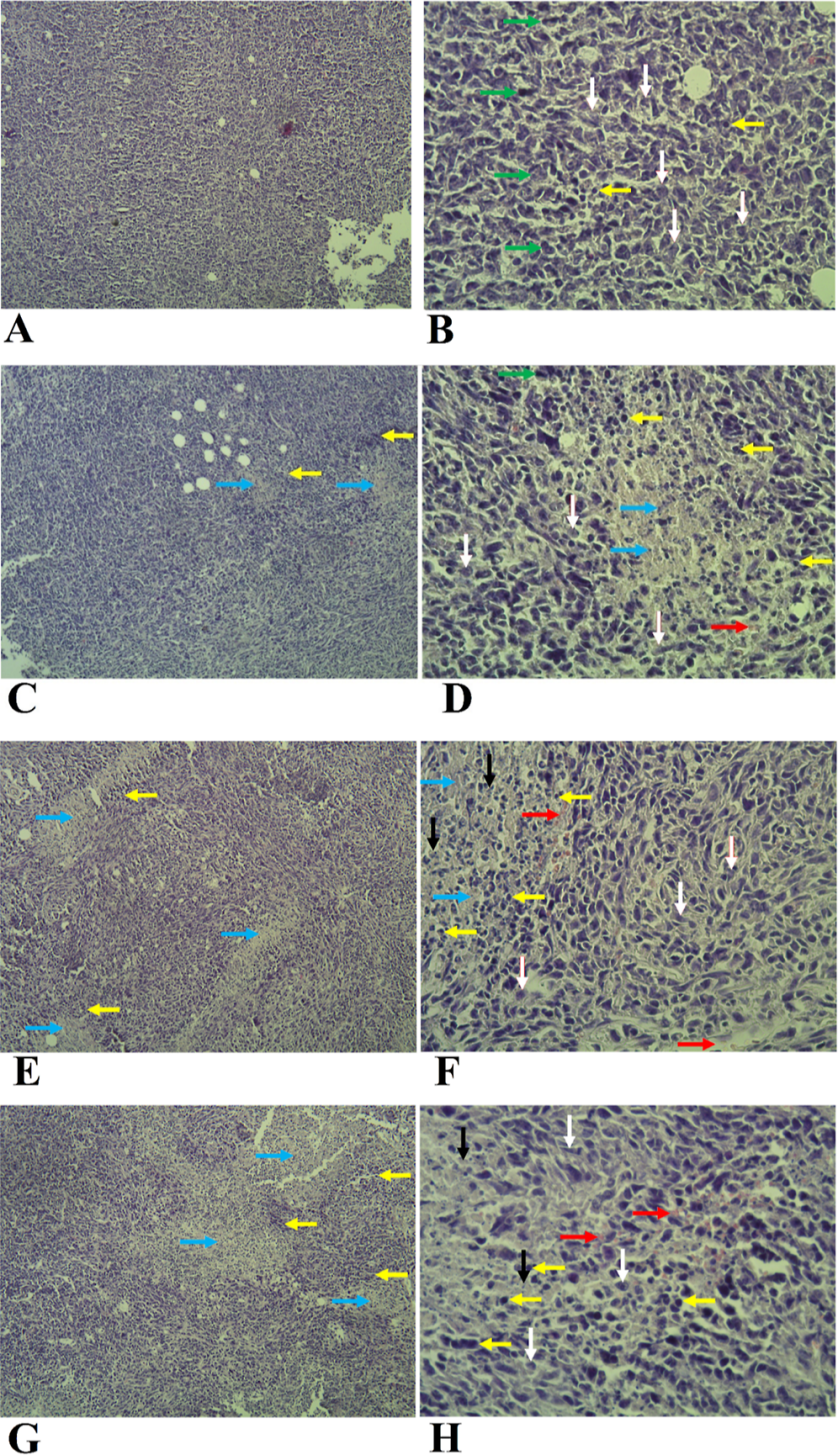
Microscopic images of H & E stained tumor tissue samples from different treatment groups: (A,B) PBS, (C,D) AuNPs, (E,F) PTX, and (G,H) FP groups. The magnification of (A,C,E, G) is 100× and the magnification of (B,D,F,H) is 400×. Arrows mark specific features such as mitosis (green) or bleeding (red), and they are detailed in the text.

Tumors obtained from animals in experimental group II were checked for changes in tissue and cell morphology by a histology analysis of stained tumor sections. In the sections from the PBS-treated group of breast tumors (Figure 11A and B), a large number of neoplastic cells are in the process of proliferation and in different stages of mitosis. Different stages of division can be seen in most of the cells present (green arrows). Tumor cells are polymorphic and undifferentiated and often have irregular and hyperchromatic nuclei as well as basophilic nuclei and little cytoplasm (white arrow). The presence of necrotic and apoptotic tissue or cellular areas is low and inconspicuous, but inflammatory cells are seen in low numbers and scattered in some areas of the tumor (yellow arrow).

In the sections from the AuNPs-treated group (Figure 11C and D), compared to the group treated with PBS, the number of neoplastic cells showing a polymorphic and undifferentiated state (white arrow) and the number of neoplastic cells in the process of mitosis are reduced (green arrow). In some parts of the tumor tissue, necrotic areas can be seen (blue arrow) covered by a rim of inflammatory cells and around which inflammatory cells are concentrated in medium numbers (yellow arrow). A very small blood accumulation can be seen in the vessels around the necrotic area (red arrow).

In the sections from the PTX-treated group (Figure 11E and F), scattered necrotic parts are found in most tissue areas (blue arrow). The extent of inflammatory cells and the number of these cells are larger than in the previous groups (i.e., PBS and AuNPs, yellow arrow), and they are more densely arranged around the necrotic area. Neoplastic cells with polymorphism and nuclear compaction are observed less frequently (white arrow), and cells undergoing the process of mitosis are very rare. Some of the cells in the necrotic area have pyknotic nuclei with karyorrhexis and compact and small cytoplasm, which is a sign of cell apoptosis (black arrow). There is some vasodilation and blood accumulation around the necrotic area (red arrow).

In the sections from the FP-treated group (Figure 11G and H), necrotic areas are larger and more numerous than in the previous groups (i.e., PBS, AuNPs, and PTX, blue arrow), and the number of inflammatory cells around these areas has also increased (yellow arrow). Neoplastic cells are associated with a decrease in number and polymorphism (white arrow) and a decrease in the mitotic process (green arrow). The cells of the necrotic area often have pyknotic nuclei with characteristic karyorrhexis and small and unclear cytoplasm, which is a sign of increased apoptosis in the cells of this area (black arrow), and the presence of severe inflammation in the surrounding area also confirms the increased apoptosis in this area. There is some blood accumulation in various tissue areas (red arrow).

In summary, the histology analysis revealed that treatments with PTX alone and FP remarkably decreased the number of proliferating neoplastic cells in contrast with PBS-treated samples. A small decrease in mitotic activity was also found in samples treated with AuNPs alone. On the other hand, the AuNP-, PTX-, and FP-treated samples exhibited a remarkably higher number of inflammatory cells as well as blood accumulation probably due to vasodilatation. The accumulation of inflammatory cells was situated around the necrotic areas. Treatments with the PTX and the FP were also characterized by the presence of apoptotic cells that were localized around the necrotic areas. Since the necrotic areas were present also in samples treated with the AuNPs that did not induce remarkable apoptosis, we suggest that the formation of necrotic areas was primarily caused by infiltration of inflammatory cells and local inflammation maybe in response to nanoparticle interaction. In the case of PTX alone, the inflammation was likely in parallel or subsequent to apoptosis induction. The individual actions of the PTX and the AuNP were then potentiated in the FP that combines both components. The summary of histology evaluation is shown in Table 3. The individual groups are graded with respect to specific criteria such as neoplastic, necrotic and apoptotic, and inflammatory cells and the amount of blood accumulation.

**Table 3:** Comparison of the Groups Based on the Results of H & E Staining According to the Different Criteria^a^

neoplastic cells	necrotic and apoptotic cells	inflammatory cells and amount of blood accumulation	groups treated by
3	0	0	BPS
2	1	1	AuNPs
1	2	2	PTX
1	3	3	FP

a The observed changes are graded from 0 to 3. Grade 0 means no change, grade 1 means slight change, grade 2 means moderate change, and grade 3 means severe.

### Assessment of AntimiR-135b Activity and Reduction of Oncogenic MiR-135b in Tumor Cells

AntimiR-135b was designed to specifically bind to oncogenic miR-135b in tumor cells and promote its degradation. To assess the effectivity of antimiR-135b cytoplasmic delivery and its ability to bind its target, we compared miR-135b levels in tumors treated with PBS and the AuNPs, PTX, or FP. The levels of miR-135b quantified with qPCR were normalized to those of internal control miR-16. Significance of the miR-135b reduction was calculated with ANOVA group comparison with the Tukey post hoc test. Since the AuNP carriers were not equally distributed throughout the entire tumor tissue, we analyzed the amount of Au in the homogenates with a GF-AAS. The amount of each homogenate used for RNA extraction was then equalized to 49 ng of Au.

**Figure 12:**
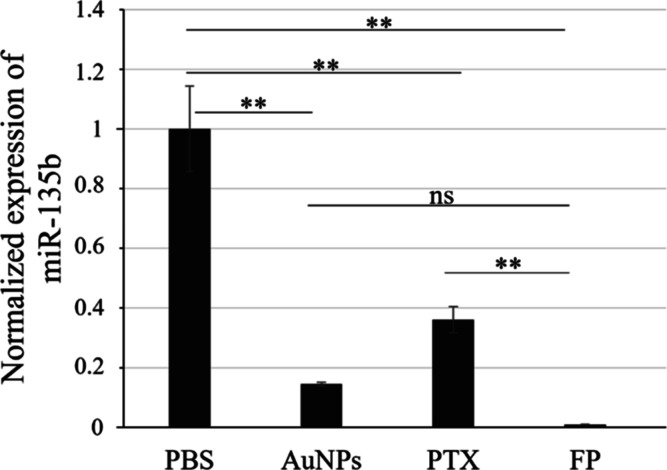
MiR-135b levels in tumors treated with FP comprising inhibitory sequence antimiR-135b designed to specifically degrade miR-135. Samples treated with PBS served as controls without effect on miR-135b levels. Values of *p* ≤ 0.01 (**) showed significant differences between groups. “ns” marks nonsignificant change.

Compared to the PBS group, a significant decrease of miR-135b was detected (Figure 12) in samples treated with FP (*p* value ≤ 0.01), AuNPs (*p* value ≤ 0.01), and PTX (*p* ≤ 0.01). We also checked the differences in miR-135b reduction among the FP and the individual components like AuNPs and PTX. We found a significantly stronger reduction of the miR-135b level after FP treatment in comparison to the solely PTX treatment (*p* value ≤ 0.01). The difference between tumors treated by solely AuNPs and tumors treated with FP was not significant (*p* value = 0.107). We suggest that FP entered tumor cells and delivered antimiR-135b to the cytoplasm. Since the antimiR-135b was able to bind and degrade the miR-135, we expect the antimiR-135b to be released from the FP and that its loading and unloading from the AuNP carriers did not affect the structure and function of the antimiR-135b molecule. These results agree with our previous studies on antimiR-135b delivery by functionalized nanodiamond carriers. We suspect a similar mechanism of nanocarrier entry and cargo released as described by our group.^22,39^ However, this assumption has to be proved since the biologically produced nanoparticles are unique type nanocarriers and their ability to transport effector RNA has not been studied yet. The qPCR also confirmed that FP has a higher efficacy in miR-135b reduction than the individual components (PTX and AuNPs). While the miR-135b reduction by PTX was expected due to its complex anticancer effect, the reduction of miR-135b by solely AuNPs was unexpected. Independently, we observed alteration of cancer-associated proteins as a response of 4T1 tumor cells to biologically produced AuNPs in our subsequent study (data not shown, article in preparation). It seems that AuNPs may induce some anticancer activity in BC cells, and the down-regulation of oncogenic miRNA-135b could be one of the effector mechanisms. More exploration in this field is necessary though to understand this phenomenon.

In this study, we prepared AuNPs using the fungal strain and conditions that we developed previously.^15,16^ Many characteristics of the AuNPs (the maximum absorption peak at 528 nm, round shape, average size of 15 nm, and zeta potential of −35 mV), produced in different batches at different times were consistent.^13−15,19,20,44^ It confirms the ability of the fungus to reliably produce similar AuNPs and obtain reproducible data.

The presented data showed that the AuNPs were able to bind to PTX, antimiR-135b, and Tf, both individually and simultaneously. The AuNPs were stable when conjugated with different cargoes, and their size stayed under 100 nm. Stability of the FP was even higher compared to that of AuNPs-PTX, which was probably due to the presence of other cargoes (i.e., Tf and RNA) contributing to the stabilization of the complex. For example, antimiR-135b with its negative charge improved the stability and overall net charge of FP (−38.84 mV in AuNPs-antimiR versus −30.2 mV in AuNPs-PTX, −35.6 mV in AuNPs-Tf, and −31.9 mV in FP).

Using the tumor-specific ligand–transferrin, Tf, for decoration of the FP led to higher specificity of the FP toward tumor cells as shown in cytotoxicity tests. Here, the 4T1 cells were more inhibited by FP than NIH/3T3 cells, which may be due to the (1) overexpression of the Tf receptor on the surface of 4T1 cells^45^ and confirming the tumor-targeting properties of FP and (2) higher growth rate of 4T1 compared to NIH/3T3 cells sensitizing the 4T1 cells to the toxic effect of FP. The second presumption was based on the observation of increased uptake and accumulation of Tf-free AuNPs in 4T1 cancer cells compared to NIH/3T3 and RAW264.7 cells that we published earlier.^46^

Final application of the FP in vivo using the 4T1 syngeneic tumor model showed important aspects of the AuNPs as drug carriers. To reduce the number of experimental animals according to the 3R strategy, Czech Animal Protection Act no. 246/1992, we performed the in vivo study with the complex FP conjugate and controls consisting of the main components (AuNPs, PTX). The FP applied peritumorally exhibited good penetration into the tumor tissue followed by inflammatory response, apoptosis, and necrosis in the tissue adjacent to application sites. At the molecular level we observed successful inhibition of target miR-135b in tumor cells. Since miR-135b is present in cytoplasm, the FP conjugate had to enter the cell and unload the antimiR-135b cargo into the cytoplasm. The exact mechanism of internalization and potential release of the AuNP-based conjugate from internalizing vesicles such as endosomes needs to be further revealed. It remains unclear if the FP stayed stable and traveled into cytosol functionalized with all decorative molecules including antimiR-135b or the molecules were at least partially released already in transport vesicles as we found in the case of nanodiamond carriers.^43^

Location of AuNPs within the cell was uncovered by TEM. The AuNPs were located in the organelles, such as the mitochondrion and ER, but not in the nuclei. This suggests that the mechanism of internalization of the biologically produced AuNPs through the plasma membrane differs from the mechanism of internalization through the nuclear membrane (which is mainly through nuclear pore complexes).^47^ The absence of AuNPs in nuclei is favorable because (1) FP must be translocated to the cytosol where target miR-135b is located and (2) it decreases the possibility of FP-mediated genotoxicity. Regarding the mean of internalization, we expect the FP entered cells via the clathrin-mediated endocytosis pathway due to Tf present on its surface.^48^ Moreover, the AuNPs (including those within the FP conjugate) were located in endolysosomes as observed by TEM. It is a question if part of the AuNP carriers escaped the endosomes and delivered antimir-135b into the cytoplasm or if the short RNA was released from the carrier and transported to the cytoplasm during the endosome maturation endolysosomes. The physicochemical properties of nanocarriers, such as size, shape, surface charge, and surface coating, as well as the amounts of individual molecules, are important for their fate in cells.^49,50^ For example, clathrin-independent endocytosis was observed in the case of folic acid, whereas clathrin-dependent and -independent endocytosis was observed in the case of folic acid-conjugated NPs.^50^ The nonbiological AuNPs were reported to be localized in the subcellular vesicles and rarely within the organelles or cytosol.^50^ On the other hand, the biologically produced AuNPs with capping agent and multiple functionalization, the FPs, used in this study were observed within organelles and cytosol. Approaches to promote the internalization of nonbiological AuNPs in the cytoplasm were made and involved conjugation of dynein-binding peptides or cell-penetrating peptides to the surface of AuNPs^51,52^ or conjugation of TAT and HA2, two viral proteins, to the surface of AuNPs.^53^

Endosomal escape seems to be a major limitation in successful drug delivery. The attempts to disrupt and bypass the endosomal pathway involved direct methods such as microinjection, direct translocation, use of bacterial pore-forming toxins or electroporation, and indirect methods such as lysis of the endosome using drugs or osmotic shock.^53^ The FP tested in the current study passed through the endosomal pathway and delivered cargo, i.e., PTX and antimiR into the cytoplasm. We are aware that Tf helps the conjugate to find target cells and enter them by endocytosis,^54^ but whether Tf helped the AuNP conjugates to overcome the endosomal barrier by passing the trans-Golgi network, then ER and finally the cytosol^55,56^ has to be analyzed in the future. We also consider passive diffusion (i.e., direct penetration)^57^ of FP into the cytosol. We expect that antimiR-135b mostly releases from the conjugate into cytoplasm due to low pH of intracellular vesicles, and this assumption is based on our experience.^57^ The release rate and fate of paclitaxel remain to be elucidated.

We have previously produced AuNPs by the biological technique and assessed the efficiency of this method.^58^ We have used biologically produced AuNPs as a drug carrier for PTX, as well as other anticancer and antibiotic drugs.^14,19,20^ There are some studies that have shown the anticancer effects of biologically produced AuNPs using microbial^59^ or plant extracts.^60^ Furthermore, there is a report on using plant-produced AuNPs in conjunction with diclofenac^61^ or doxorubicin (DOX).^62^ However, this is the first time we have been able to conjugate the AuNPs with different cargoes simultaneously, producing a novel bionanocarrier for targeted cancer therapy. Previous attempts have been made to conjugate single drugs to nonbiologically produced AuNPs. For example, Paciotti et al. conjugated PTX to chemically produced AuNPs using chemical reagents and processes such as thiolations.^63^ One study demonstrated that conjugating the microtubulin inhibitor, DM1, to nonbiologically produced AuNPs increased drug cytotoxicity and half-life in hepatocellular carcinoma.^64^ Another study using kaempferol against A549 lung cancer cells showed higher toxicity against cancer cells compared to normal cells.^65^ In the case of multifunctional AuNPs, research has been conducted using porous silicon nanoparticles and rod-shaped AuNPs. Afatinib (Afb) was used to target HER-2 and EGFR and release DOX and rapamycin drugs with acceptable in vitro and in vivo results.^66^ Many studies have been conducted on conjugating drugs and other molecules for targeted therapies using nonbiological AuNPs. These studies require the use of chemical reagents to link the AuNPs to specific cargoes as well as multiple steps and washings, which are eliminated when using biologically produced AuNPs. The novelty of the current study lies in delivering one missing piece of information on conjugation and characterization of biologically produced AuNPs with multiple effector molecules, such as anticancer drugs and antimicro RNA, which specifically target cancer cells.

## Conclusions

In the current study, we prepared and successfully tested a multifunctional drug delivery agent based on biologically produced AuNPs. AuNPs were able to conjugate directly with various cargoes. This is due to their “capping agents”, which are likely polypeptides or amino acids that can bind to different types of cargoes due to their polar properties. The high load capacity of biologically produced AuNPs for various molecules makes them a good candidate for transporting drugs or genes into target cells. In this study, we showed that the FP containing PTX, Tf, and antimiR-135b could reduce cell survival better than the same concentration of PTX as the control in vitro. The results confirmed the ability of FP to penetrate the tumor, induce cell death and inflammation, and regulate gene expression in vivo. It seems that AuNPs serve as multifunctional carriers for targeted therapy, and detailed investigation with respect to internalization of the biologically produced AuNPs is needed, and we plan to perform it in our future studies.

## Supporting Information Available

Data from measurements of zeta potential and hydrodynamic parameter; color change documentation after NP conjugation with cargo; standard curves of different cargoes for load evaluation; detection chromatogram of PTX; assay values to identify toxicity IC_50_, and particle size distributions revealed by DLS (PDF)
