# Supplementary material for: Bioproduced Nanoparticles Deliver Multiple Cargoes via Targeted Tumor Therapy In Vivo
Source: ACS Omega. 2024 Jul 23;9(31):33789–804. doi: 10.1021/acsomega.4c03277 (PMC11307291; doi:10.1021/acsomega.4c03277)

Supplementary information

Bio-produced nanoparticles deliver multiple cargo *via* targeted tumor therapy *in vivo*

Parastoo Pourali[1]†, Eva Neuhöferová[1]†, Volha Dzmitruk[2], Milan Svoboda[3], Eva Stodůlková[1], Miroslav Flieger[1], Behrooz Yahyaei[4, 5] & Veronika Benson[1,6*]

[1]Institute of Microbiology, Czech Academy of Sciences, Czech Republic

[2]Center of Molecular Structure, Institute of Biotechnology, Czech Academy of Sciences, Czech Republic

[3]Institute of Analytical Chemistry, Czech Academy of Sciences, Brno, 602 00, Czech Republic;

[4]Department of Medical Sciences, Shahrood Branch, Islamic Azad University, Shahrood, Iran;

[5]Department of Medical Sciences, Biological Nanoparticles in Medicine Research Center, Shahrood Branch, Islamic Azad University, Shahrood, Iran;

[6] Faculty of Health Studies, Technical University of Liberec, 46001 Liberec, Czech Republic

†Both with the same contributions and preferably as first authors.

[*]Email: benson@biomed.cas.cz

Table S1.

Hydrodynamic diameter and zeta potential of default AuNPs and AuNPs conjugated with different cargoes measured by a Zetasizer. Individual measurements used for statistical analyses.

**Tested material**	**Parameter / Repeat**	1	2	3	4	5	**Mean**	**SD**
**AuNPs**	**Hydrodynamic diameter (nm)**	11	13	15.2	13	13	**13.04**	**± 1.33**
	**Zeta potential (mV)**	-35.1	-34.64	-35.7	-35.8	-37.7	**-35.8**	**± 1.17**
**AuNPs-Tf**	**Hydrodynamic diameter (nm)**	68.7	59.1	64	68.7	79.9	**68.1**	**± 7.71**
	**Zeta potential (mV)**	-35.5	-34.1	-33.6	-37.3	-35.6	**-35.2**	**± 1.45**
**AuNPs-PTX**	**Hydrodynamic diameter (nm)**	59.1	59.1	79.9	79.9	68.7	**69.3**	**±10.41**
	**Zeta potential (mV)**	-27.6	-29.7	-31.5	-30.7	-31.7	**-30.2**	**± 1.67**
**AuNPs-antimiR**	**Hydrodynamic diameter (nm)**	28	38	32	32	32	**32.4**	**± 3.2**
	**Zeta potential (mV)**	-38.1	-42.7	-37.2	-38.1	-38.1	**-38.84**	**± 1.97**
**FP**	**Hydrodynamic diameter (nm)**	68.7	63	43.7	37.6	68.7	**54.2**	**± 14.10**
	**Zeta potential (mV)**	-28.3	-32.1	-33	-32.8	-33.4	**-31.9**	**± 2.08**

Figure S1.

A) Color change observation after conjugation of AuNPs with different cargoes.

B – D) Standard curves of Tf, antimiR-135b, and PTX that served for the load estimate of individual component within AuNP conjugates.

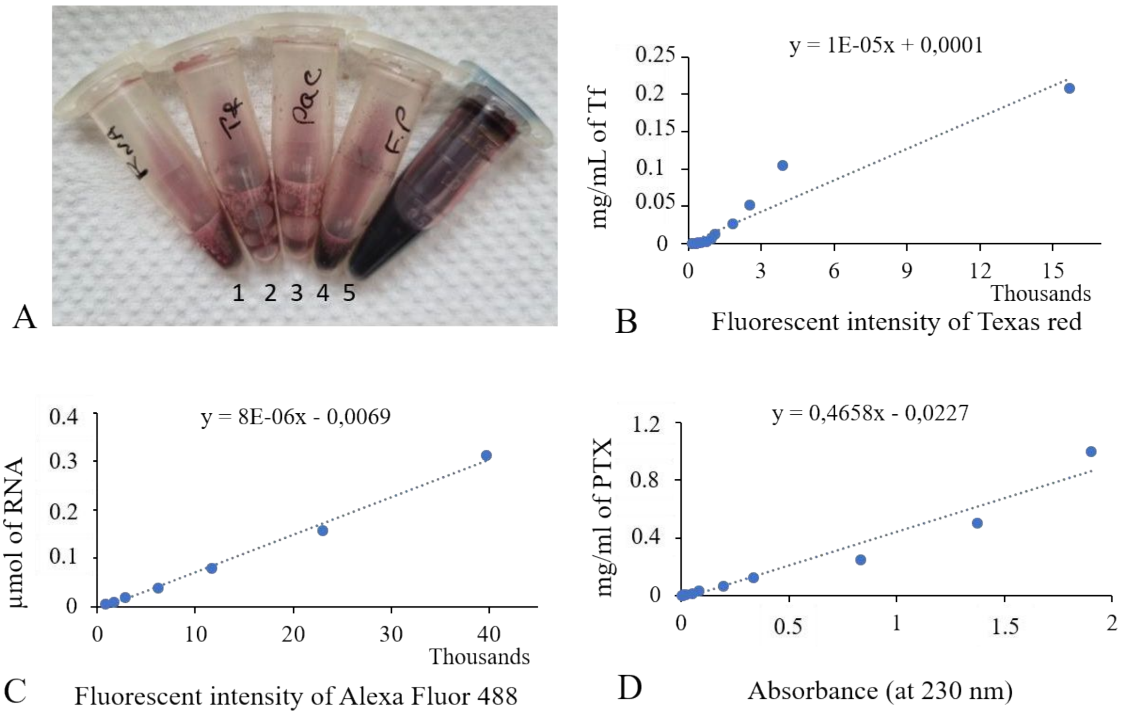

Figure S2.

A) Determination of free PTX remaining in supernatant after preparation of nanoparticles (starting concentration of PTX 107 µg/ml, blue line). Chromatogram of standard solution of PTX (20 µg/ml, black line) is given for the comparison.

B) Determination of free PTX remaining in supernatant after preparation of nanoparticles (starting concentration of PTX 545 µg/ml, black line). Chromatogram of standard solution of PTX (20 µg/ml, blue line) is given for the comparison.

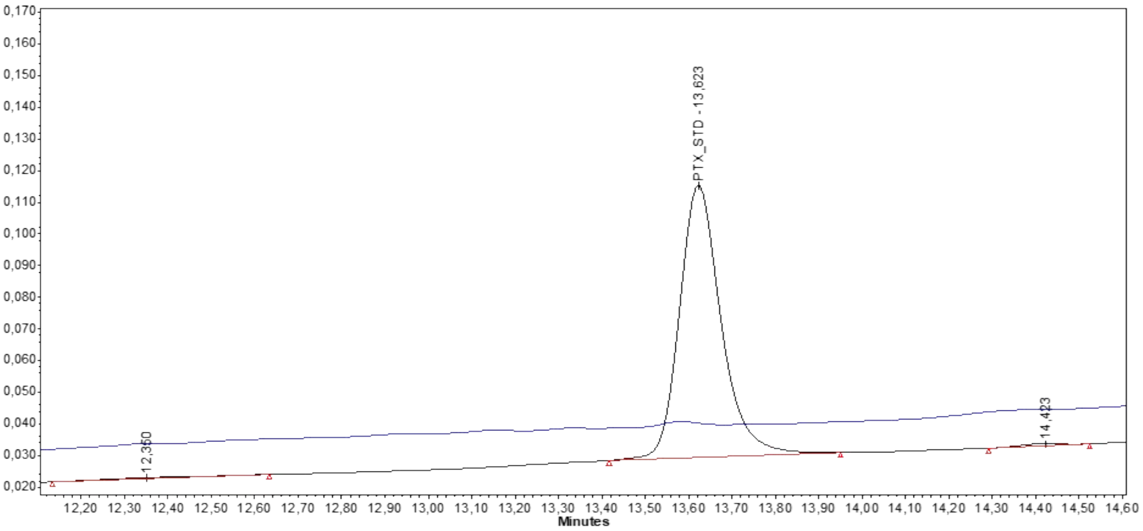

A

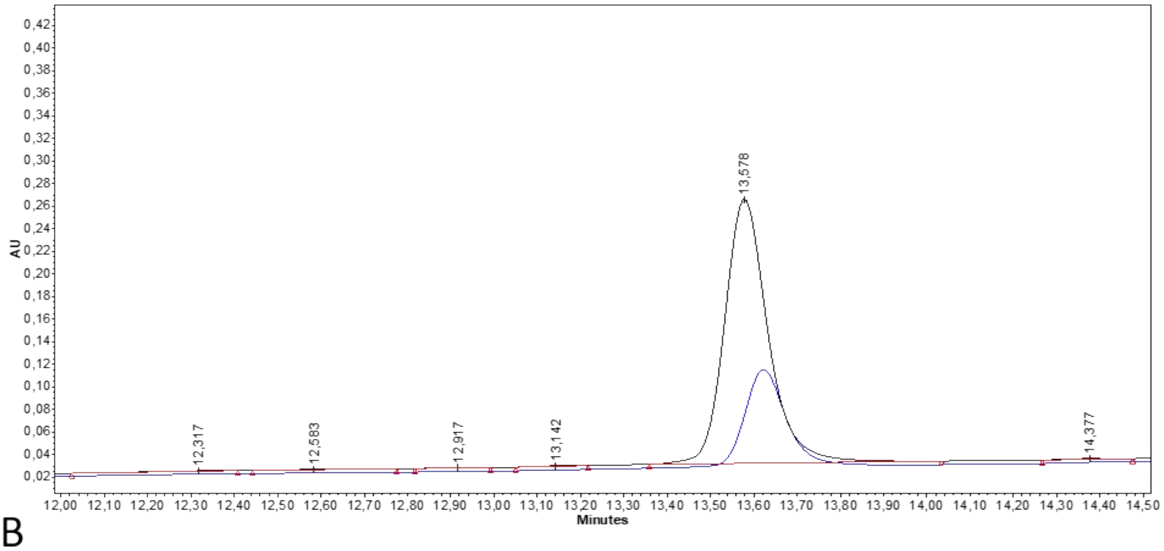

B

Table S2.

The calculated averages of the replicates obtained from the difference between absorbance at 570 nm compare to reference wavenumber (i.c.,630 nm). IC50 for each sample is determined and showed with * sign. The rows are: A1-12) FP in 4TI cell line, BI-B9) free PTX in 4T1 cell line, B10-12) 4T 1 cells without any treatment, C) FP in NIH/3T3 cell line, DI-D9) free PTX in NIH/3T3 cell line, D10-12) NIH3T3 cells without any treatment.

Row/well	1	2	3	4	5	6	7	8	9	10	11	12
A	0.238	0.0611	0.0734	0.1303	0.3774*	0.6575	0.7899	0.800	0.7021	0.742	0.6934	0.7623
B	0.1258	0.2558*	0.6601	0.7836	0.7252	0.8438	0.9318	0.801	0.8214	0.830	0.830	0.830
C	0.1902	0.3280	0.5210*	0.8103	1.1895	1.2285	1.3851	1.533	1.1949	1.327	1.156	1.216
D	0.14995	0.5037*	1.0884	1.34465	1.3197	1.3563	1.2812	1.4356	1.1990	1.2121	1.2121	1.2121

Figure S3.

Particle size distribution by intensity percentage of AuNPs and AuNPs-conjugated samples. Size is presented as particle diameter (nm).

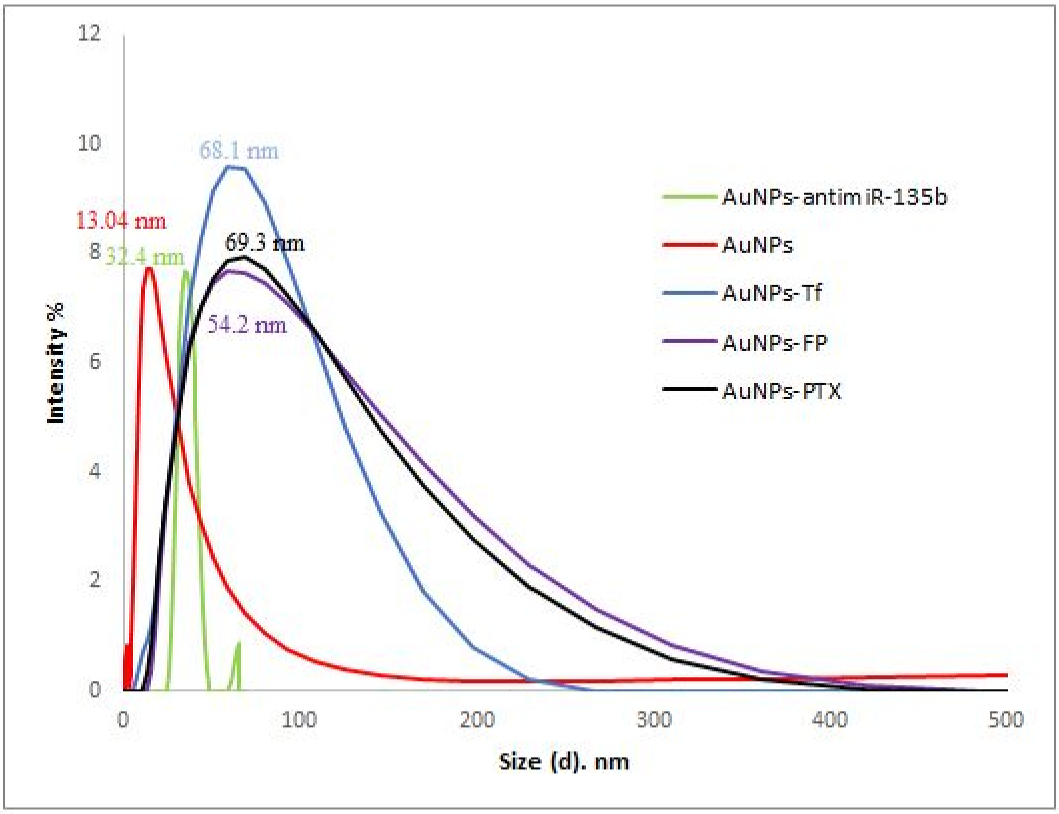

Supplement: Supplementary file 1 — ao4c03277_si_001.pdf [file ao4c03277_si_001.pdf]
